# Targeted axillary dissection in breast cancer patients: a systematic review and meta-analysis

**DOI:** 10.1038/s41523-026-00984-3

**Published:** 2026-06-05

**Authors:** Mohammed. N. Abdelaziz, Sagad O. O. Mohamed, Mohammed Hesham Nagi, Ibrahim Saleh Alawadi, Youstina Mohsen, Sohaila Essam Ibrahim, Radwa M. Abdelsattar, Asmaa. N. Abdelaziz, Mohamed Yasser, Sherif Wael, Hend A. Abdelgawad, Magdy Shehab, Khaled M. Abdelwahab, Omar Hamdy

**Affiliations:** 1 Faculty of Medicine, Mansoura University Hospital, Mansoura, Egypt; 2 University of Khartoum, Khartoum, Sudan; 3 Faculty of medicine, Kasr Alainy, Cairo University, Cairo, Egypt; 4 Surgical Oncology Department, Oncology Centre, Mansoura University, Mansoura, Egypt

**Keywords:** Cancer, Medical research, Oncology

## Abstract

Targeted axillary dissection (TAD) is an innovative approach for axillary staging in breast cancer patients with initially node-positive disease that converts to clinically node-negative status after neoadjuvant therapy. Optimal marking and localization techniques remain undetermined. This systematic review and meta-analysis searched PubMed, Scopus, Cochrane Library, and Web of Science through April 2025, including 59 observational studies on TAD (marked/clipped node removal plus sentinel lymph node biopsy) in such patients. Primary outcomes were identification rate, false-negative rate (FNR), concordance, and diagnostic accuracy. Using R software with random-effects models and logit transformation, TAD achieved a pooled identification rate of 95.1% (95% CI: 93.2%–96.5%). Pooled FNR was 6.37% (95% CI: 5.02%–8.04%; *I*² = 0.0%), with overall diagnostic accuracy of 94.68% (95% CI: 91.70%–96.63%). Targeted and sentinel nodes concorded in 73.34% (95% CI: 69.58%–76.79%). Subgroup analyses showed comparable performance across marking (clip, carbon, magnetic, radioactive seeds) and localization techniques (wire-guided, radioactive, magnetic, ultrasound-guided), with no significant differences. TAD offers high identification rates, low FNRs, reliable staging, and acceptable accuracy, with consistent results enabling implementation using locally available technologies.

## Introduction

The status of axillary lymph nodes remains the strongest prognostic indicator in breast cancer patients, and axillary staging remains one of management’s most important tools^1^. It has evolved over the years from radical axillary lymph node dissection (ALND) to even “no surgery”, for some selected patients^2–7^. Today, the axillary staging question in patients who are node-positive and become node-negative after neoadjuvant therapy (NAT) continues to provide very interesting areas of research and raises some issues with contradictory points of view^8–11^.

Essentially, sentinel lymph node biopsy (SLNB) has become recognized as the standard of care for node-negative early breast cancer, displacing ALND^12,13^, and in fact, has even been deemed harmful overtreatment in non-indicated patients^14^. In contrast, because of oncological safety concerns, the application of the SLNB paradigm to breast cancer patients who underwent NAT occurred a bit later, and only after it was established to be oncologically sound. Concerns were raised with this category of patients regarding the response of obstructed lymphatics to chemotherapy, among the variable responses of lymph nodes, and the influence of non-sentinel nodes^15,16^. These issues were discussed as facts, particularly when the key studies published reported prohibitively high false-negative rates (FNRs) for SLNB in this patient cohort^17–19^.

However, this situation did not persist long. The first issue that was resolved was the SLNB in node-negative breast cancer patients who underwent NAT, as this group of patients with clinically negative axillary nodes before and after NAT did not need any procedure beyond the standard SLNB^20–22^. In contrast to the initial group, the group of patients with node-positive disease who became node-negative is the reason that leading studies like SN-FNAC^23^, SENTINA^24^, and the ACOSOG Z1071^25^ established the fact of a high FNR, and contributed to designing plans to mitigate the effect of the factors that contribute to this FNR (node count, number of tracers used, and if the malignant nodes were marked before NAT). Subsequent studies confirmed that these are the strategies to reduce this FNR, and finally^26^, those recommendations were integrated into the management guidelines^27^.

As a result, many studies began evaluating the idea of axillary lymph node marking prior to NAT beginning in 2016^28^. This has been more commonly referred to as “targeted axillary dissection (TAD),” meaning excision of the marked lymph node and the sentinel lymph node after NAT. Many studies have found that TAD decreases false negative rates and is much less morbid than ALND^29^. Questions of “How do we mark the node?” and “How do we localize the marked node pre-operatively?” have been a focus of ongoing research over the last decade to arrive at a standardized TAD tool or at least substitutes that could be fitted with whatever tools might be available in any one health institution anywhere in the world^29^. This study, therefore, aimed to define optimal TAD by directly comparing the efficacy of various localization strategies in patients with breast cancer who were node-positive at presentation but clinically node-negative after neoadjuvant therapy and provide evidence-based recommendations regarding how to choose among localization strategies to maximize oncologic safety and limit surgical morbidity.

## Results

### Study selection

**Fig. 1:**
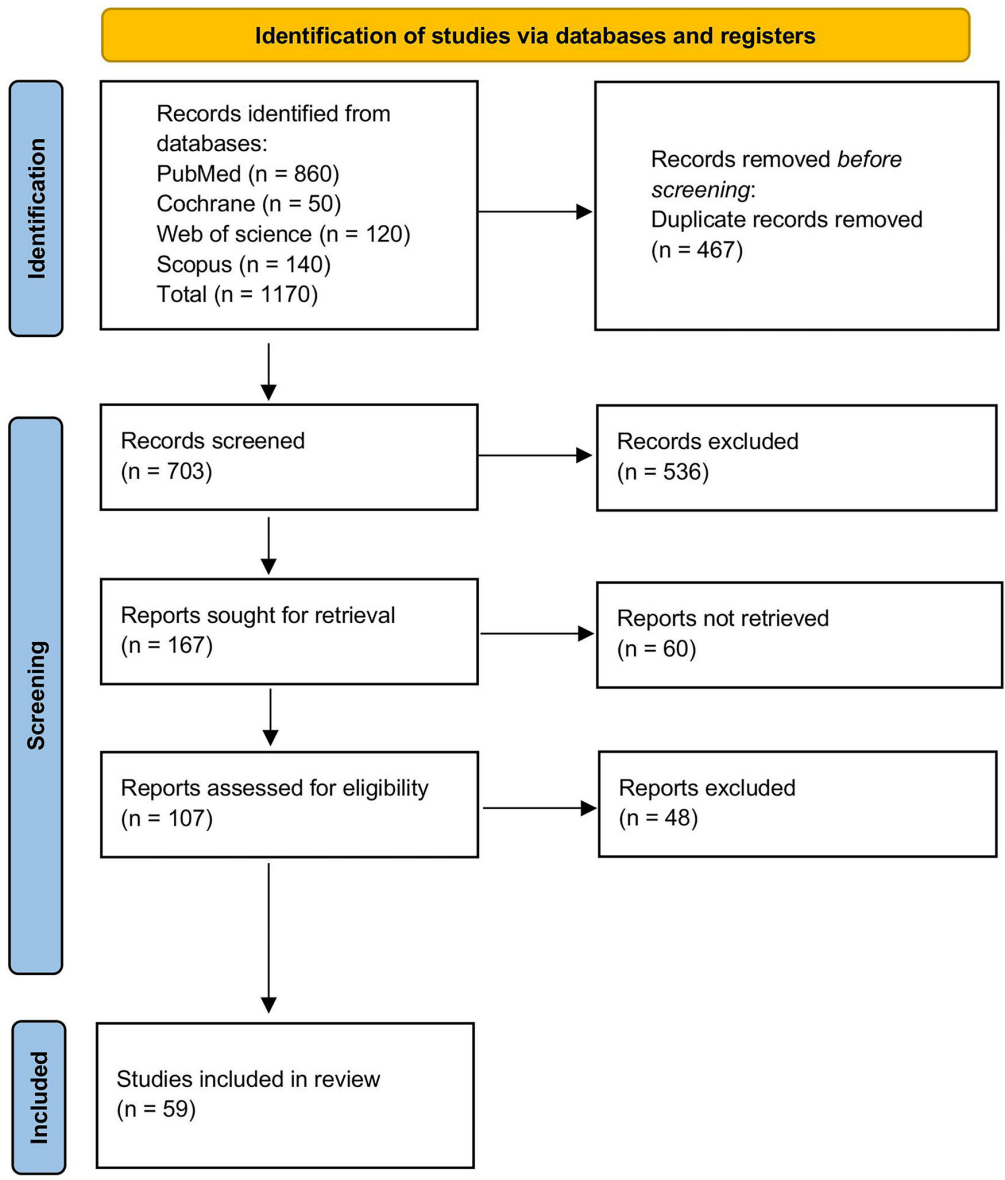
PRISMA flow chart illustrates studies selection. PRISMA flow chart illustrates the process of study selection for the systematic review. The diagram details the identification phase with records from databases: PubMed (*n* = 860), Cochrane (*n* = 50), Web of Science (*n* = 120), and Scopus (*n* = 140), totaling 1170 records. Duplicate records were removed before screening numbered 467. Screening involved 703 records, excluding 536; 167 reports were sought, but 60 were not retrieved. Eligibility assessment of 107 reports excluded 48, leaving 59 studies included in the review.

A systematic search across four databases (PubMed, Web of Science, Cochrane Library, and Scopus) yielded 1170 records. Using EndNote 21 for reference management and Rayyan for blinded screening, after removing duplicates, 703 unique records were screened by title and abstract. Of these, 167 full-text articles were sought for retrieval, and 107 were successfully assessed for eligibility as detailed in the PRISMA flow diagram. We finally included 59 observational studies conducted across different countries. (Fig. 1).

### Characteristics of included studies

Fifty-nine studies were incorporated into this systematic review/meta-analysis between 2015 and 2025. In general, the evidence base was dominated by prospective designs (36/59), with the rest comprising largely retrospective cohorts (22/59) and one pilot study. These studies spanned multiple areas, most prominently in the United States and Spain, followed by China, Turkey, and Germany, and with other contributions from other European and non-European countries. The average patient age was in the early to mid-50s (overall mean of reported study means = approximately 51.9 years). Most of the cohort included patients presenting with clinically node-positive disease at diagnosis (mainly cN+/cN1, but some studies also include broader cN1-2 or cN1-3 populations), reflecting standard clinical indication for TAD after neoadjuvant therapy.

Most studies reported some neoadjuvant systemic therapy, which was mostly chemotherapy with anti-HER2 targeted therapy as the most common co-adjuvant therapy. Endocrine therapy was not so common and was reserved for select hormone receptor−positive cases. Histopathological reports, when given, predominantly cited invasive ductal carcinoma, with a minority of notations of invasive lobular carcinoma and other histologies. Reporting of molecular subtype was typically in terms of hormone receptor−positive/HER2-negative, HER2-positive, and triple-negative phenotypes (usually within the same cohort). In terms of the TAD technique, a clip to mark the target lymph node was the most common technique. Other marking modalities would have included carbon tattooing, magnetic markers, radioactive seeds, and, less frequently, reflectors or mixed/unspecified modalities. Methods of localization at the time of surgery were quite heterogeneous; wire-guided localization and radioactive localization were the most common, closely followed by mixed approaches and visual methods. Additional modalities mentioned include magnetic localization, reflector-based localization, ultrasound guidance, and carbon-specific localization.

SLNB mapping methods varied, although most studies employed the dual tracer method (radioisotope+blue dye) (34/59) or radioisotope alone (10/59), with fewer using only blue dye (7/59) or fluorescence/ICG-based approaches (2/59). In terms of follow-up reporting, it was rather inconsistent, but among studies that reported a median/typical follow-up duration, it was generally short to intermediate (reported values were commonly around 2 years, with a median of 22 months among those reporting follow-up). Procedure-related complications were infrequently reported. Axillary/local recurrences were rare (0–1.8%), and complications remained low (0% in ~20 studies; up to 15.7% pain/1.2% allergy, 14.3% seroma). The summary of the Included Studies and Patients’ Characteristics was presented in Table 1. Detailed characteristics of the included studies were explained in the supplementary table S1.

**Table 1:** Summary of Included Studies and Patients’ Characteristics

Study ID	Location	Study design	Sample size	Age (mean ± SD)/median (IQR)	Inclusion criteria	Marking technique	Localization Technique	SLNB Method	Overall quality assessment score (/9)
Acea-Figueira et al.^51^	Spain	Prospective study	81	53.3 ± 11.5	Women over 18 with biopsy-confirmed cN1 breast cancer who received primary systemic therapy and had clip-marked metastatic lymph nodes	Clip	Wire	Tc-99m + blue dye	4
Aguirre et al.^52^	Spain	Prospective study	80	54.7 ± 11.4	Women with invasive breast cancer treated with NAC	Radioactive	Radioactive	Dual method	4
Alarcón et al.^53^	Spain	Prospective study	103	54.4 ± 12.7	Biopsy-proven node-positive breast cancer patients who received NAT	Clip	Wire	Tc-99m	4
Aragon-Sánchez et al.^54^	Spain	Prospective study	32	50.8 ± 3.4	Women with biopsy-confirmed cT1–T3N1 breast cancer who underwent clip placement prior to neoadjuvant chemotherapy and received TAD after clinical axillary response	Clip	Mixed	Tc-99m + blue dye	7
Balasubramanian et al.^55^	United Kingdom	Retrospective study	47	48.75 ± 11.5	Women with primary breast cancer and biopsy-proven axillary metastases who had an excellent nodal radiological response following NAC	Clip	Wire	Tc-99m + blue dye	8
Barry et al.^56^	United Kingdom	Prospective database study	221	54.0 ± 13.2	Patients requiring axillary nodal marking	Magnetic	Magnetic	Dual tracer (radioactive + blue dye)	8
Beniey et al.^57^	Canada	Retrospective study	35	50.75 ± 11.17	Women aged ≥18 with biopsy-proven axillary metastases who achieved complete clinical axillary response after NAC and subsequently underwent TAD	Clip	Radioactive	Tc-99m + blue dye	4
Boniface et al.^36^	Sweden and Germany	Prospective study	194	NR	Women with cN1 breast cancer who converted to cN0 after neoadjuvant systemic therapy	Carbon	Visual	Tc-99m + blue dye	4
Boyle^41^	USA	Retrospective study	44	47.75 ± 3.4	Women with stage II-III, node-positive breast cancer who became clinically node-negative after NAC	Clip	Mixed	Tc-99m + blue dye	5
Castillo et al.^58^	Spain	Retrospective study	54	NR	Women with cT1-4b cN1 infiltrating breast cancer who converted to ycN0 after NAC	Clip	Radioactive	Tc-99m	5
Caudle et al.^28^	USA	Prospective study	208	51.25 ± 11.1	Women with biopsy-proven node-positive breast cancer and a clip placed in the metastatic node	Clip	Radioactive	Tc-99m ± blue dye	5
Chen et al.^59^	China	Prospective study	77	47.5 ± 8.7	Women aged 18–70 with cT1–3N1M0 breast cancer and pathologically confirmed lymph node metastasis	Clip	Visual	Tc-99m + blue dye	5
Dashevsky et al.^60^	USA	Retrospective study	28	53.25 ± 14.7	Women with locally advanced breast cancer and lymph node involvement confirmed on pre-NAC core biopsy	Clip	Wire	Tc-99m + blue dye	5
Diego et al.^61^	United States	Retrospective study	30	52.76 ± 10.05	Women with biopsy-proven node-positive breast cancer who converted to cN0 after NAC and had a clip placed in the metastatic node prior to treatment	Clip	Radioactive	Tc-99m + blue dye	5
Dilege et al.^62^	Turkey	Pilot study	15	45.5 ± 12.07	Women with clinically node-positive breast cancer who underwent clip placement before NAC and subsequent SPECT/CT to guide TAD	Clip	Radioactive	Tc-99m + blue dye	5
Dostalek et al. ^63^	Czech Republic	Retrospective study	62	52.9 ± 15.2	Women with T1-2 invasive breast cancer and 1–3 suspicious axillary lymph nodes who were planned for NAC followed by surgery with carbon tattooing of the marked nodes	Carbon	Visual	Tc-99m	5
Dux et al.^64^	USA	Retrospective study	292	51.33 ± 13.41	Women with cTis-T4N1M0, biopsy-proven node-positive breast cancer	Clip	Mixed	Tc-99m + blue dye	6
Flores-Funes et al.^65^	Spain	Prospective study	60	53.9 ± 10.5	Women with cT1-3 cN1 M0 infiltrating breast adenocarcinoma who were responsive to NAC and candidates for post-treatment surgery	Clip	Mixed	Tc-99m	6
Gallagher et al.^66^	USA	Prospective study	86	55 ± 11.86	Women aged ≥18 with T1-2/N1-3 invasive breast cancer and biopsy-proven nodal disease	Reflector	Reflector	NR	6
García-Novoa et al.^67^	Spain	Prospective study	42	51.3 ± 12.3	Women aged ≥18 with infiltrating breast carcinoma and pathologically confirmed N1 disease who underwent NAC	Clip	Wire	Tc-99m + blue dye	6
Gurleyik et al.^68^	Turkey	Prospective study	64	45.6 ± 9.63	Women with cT1-3 N1 M0 breast cancer, biopsy-confirmed axillary metastasis, and pre-NAC clip placement	Clip	Wire	Blue dye	6
Hartmann et al.^69^	Germany	Prospective study	30	50.25 ± 12	Women with cT1-3N1-3M0 invasive breast cancer, confirmed axillary metastases, and treated with primary systemic therapy	Clip	Wire	Blue dye + radiocolloid	6
Kaya et al.^42^	Turkey	Retrospective study	83	50.5 ± 9.2	Women with cN1-N2 breast cancer receiving NAC	Clip	Wire	Blue dye	5
Kim et al.^70^	South Korea	Prospective study	28	49 ± 9.4	Women with cytologically confirmed axillary metastases who underwent pre-NAC clip placement and post-NAC charcoal tattooing	Clip	Carbon	Tc-99m + blue dye	6
Kuemmel et al.^71^	Germany	Prospective study	473	53.25 ± 9.48	Women with T1-4 M0, biopsy-proven node-positive breast cancer	Clip	Mixed	Tc-99m ± blue dye	8
Kuemmel et al.^31^	Germany	Prospective study	199	51.75 ± 9.7	Women aged ≥18 with cN+ non-metastatic early breast cancer	Clip	Wire	Tc-99m ± blue dye	4
Laws et al.^72^	United States	Retrospective study	57	51.25 ± 9.38	Women with biopsy-proven node-positive breast cancer, a pre-NAC clip, and subsequent TAD using a wireless non-radioactive localizer	Clip	Mixed	Tc-99m + blue dye	5
Laws et al.^73^	USA	Retrospective study	191	48 ± 3.3	Women with non-metastatic cN1 breast cancer treated with NAC who had ypN0 status after TAD or SLNB	Clip	Radioactive	Tc-99m + blue dye	4
Loveland-Jones et al.^74^	USA	Prospective study	110	54.75 ± 10.5	Women aged ≥18 with cT0-4 N1-3c breast cancer planned for NST	Clip	NR	Tc-99m ± blue dye	9
Manuel et al.^75^	Spain	Prospective study	30	58 ± 8.8	Women with cN1 disease, a pre-NAC clip, and ≤2 suspicious nodes who showed radiologic response	Clip	Radioactive	Tc-99m	4
Martínez et al.^76^	Spain	Prospective study	29	54.5 ± 11.8	Women with biopsy-proven axillary metastases, a pre-NAC clip, and preoperative Magseed localization	Clip	Magnetic	Tc-99m	8
Martínez et al.^77^	Spain	Prospective study	81	50.7 ± 12.7	Women with cT1-3 cN1 breast cancer and 1–4 suspected nodes indicated for NAST	Magnetic	Magnetic	NR	7
Munck et al.^78^	Denmark	Retrospective study	543	51.25 ± 10	Women with biopsy-proven axillary metastases who received ≥4 NAC cycles and underwent two-step TAD with coil marking	Clip	Mixed	Blue dye + radiocolloid	6
Munck et al.^50^	Denmark	Retrospective study	142	52.5 ± 10.7	Danish women with biopsy-proven axillary metastases who received ≥4 NAC cycles and TAD with I-125 seed marking	Radioactive	Radioactive	Tc-99m + blue dye	6
Muslumanoglu et al.^79^	Turkey	Retrospective study	501	48.5 ± 9.6	Women aged >18 with cT1-4 cN1-3 M0 disease who converted to node-negative after NAC and underwent SLNB or TAD	Clip	Mixed	Tc-99m ± blue dye	6
Patel et al.^80^	USA	Prospective study	66	54 (29 - 71)	Women with newly diagnosed breast cancer and suspicious axillary lymph nodes	Carbon	Visual	Dual agent	6
Pfob et al.^40^	Germany	Retrospective study	2698	53 (33 - 76)	Women with cN+ cM0 breast cancer who received neoadjuvant therapy before their first surgery	NR	NR	Tc-99m	9
Pinto et al.^81^	Portugal	Prospective study	31	60.4 ± 14.4	Women with cT1-3 cN1 M0 disease scheduled for NAC	Mixed	Mixed	Technetium sulfur colloid + blue dye	6
Pinto et al.^82^	Portugal	Prospective study	37	NR	Women with biopsy-proven cN1 disease who converted to ycN0 after NAT	Clip	US	ICG and blue dye	6
Plecha et al.^83^	USA	Retrospective study	107	51.75 ± 10.12	Women with core needle biopsy-confirmed axillary metastases	Clip	Wire	Tc-99m	6
Porpiglia et al.^84^	Italy	Retrospective study	49	54.9 ± 10	Women with clinically/radiologically suspicious axillary nodes pre-NAC	Carbon	Visual	Tc-99m + blue dye	6
Reitsamer et al.^85^	Austria	Prospective study	40	56 (33 - 80)	Women with biopsy-proven axillary metastases, a pre-NAC clip, and scheduled for TAD with Magseed and technetium-based SLNB	Clip	Magnetic	Tc-99m	9
Rella et al.^86^	Italy	Prospective study	72	53.5 ± 12.06	Women with non-metastatic, node-positive breast cancer, a pre-NAC clip, and no prior axillary surgery	Clip	Radioactive	Blue dye	6
Sierra et al.^87^	Spain	Prospective study	74	51.7 ± 10.08	Women with cT1-3 N1 M0 breast cancer and a pre-NAST clipped lymph node	Clip	Wire	Tc-99m	6
Simons et al.^88^	Netherlands	Retrospective study	139	58.89 ± 14.22	Women with biopsy-proven cN+ breast cancer who underwent NST followed by combined MLN and SLN excision	Mixed	Mixed	Radioactive tracers ± blue dye	6
Simons et al.^89^	USA	Prospective study	50	55 ± 10.7	Women with biopsy-proven node-positive breast cancer, a pre-NAC clip, and post-NAC magnetic seed TAD	Clip	Magnetic	Tc-99m + blue dye	6
Siso et al.^90^	Spain	Prospective study	46	52 ± 11	Women with cytologically confirmed axillary metastases, a pre-NAT clip, and planned for IOUS-guided excision, SLNB, and ALND	Clip	US	Tc-99m + blue dye	6
Siso et al.^37^	Spain	Prospective study	235	53.75 ± 11.76	Women with cT0-4 cN1 breast cancer, a clip in the positive node, and treatment with NST	Clip	US	Tc-99m ± blue dye	6
Spautz et al.^91^	Brazil	Prospective study	123	53.75 ± 10.2	Women with cT1-4 cN1-2 M0 disease who underwent pre-NAC confirmation and carbon tattooing of axillary metastases	Carbon	Visual	Blue dye	6
Sun et al.^92^	Spain	Prospective study	455	48.8 ± 11.5	Women with biopsy-proven axillary metastases who underwent NAC, radar reflector localization, and TAD	Clip	Reflector	Tc-99m + blue dye	6
Sutton et al. ^93^	USA	Retrospective study	29	52.3 ± 10.8	Women with cT0-4 N1-3 M0 disease who converted to node-negative after NAC and underwent SLNB	Clip	Visual	Tc-99m + blue dye	5
Taj et al.^94^	USA	Retrospective study	80	53.5 ± 3.5	Women with biopsy-proven axillary metastases, a pre-NAT clip, who converted to cN0 and underwent preoperative localization and SLNB	Clip	Reflector	Tc-99m + blue dye	6
Weinfurtner et al.^95^	USA	Retrospective study	147	53.25 ± 5.58	Patients who underwent SAVI SCOUT radar reflector localization of a biopsied axillary lymph node.	Clip	Reflector	Tc-99m	6
Weiss et al.^96^	USA	Prospective cohort study	238	57.5 ± 12.5	Patients who were treated with NAC and surgery	Clip	Radioactive	Dual tracer	5
Winder et al.^97^	Australia	Retrospective study	38	50 (24 - 84)	Patients who had cT1-3 N1-2 M0 disease, a pre-NAST clip, and converted to node-negative before TAD	Clip	Radioactive	Blue dye	8
Wu et al.^98^	China	Prospective study	92	48.25 ± 9.8	Women aged 18-70 with cT1-3 N1-2 M0 disease and histologically confirmed nodes planned for NAC	Clip	Wire	Tc-99m + blue dye	6
Wu et al.^99^	China	Prospective study	322	46.75 ± 8.1	Women with biopsy-proven cN1-3 breast cancer receiving NAC	Clip	Wire	Tc-99m ± blue dye	4
Yang et al.^100^	China	Prospective study	38	45.67 ± 37.23	Women with biopsy-proven node-positive breast cancer who underwent pre-NAT clip and carbon nanoparticle localization, followed by TAD and dual-tracer SLNB	Clip	Carbon	ICG + blue dye	5
Yousri et al.^45^	Egypt	Prospective study	30	52.75 ± 7.73	Women with clinically node-positive axilla planned for NAC	Carbon	Visual	Blue dye	8

*ALND* axillary lymph node dissection, *cN* clinical nodal stage, *cT* clinical tumor stage, *ICG* indocyanine green, *IQR* interquartile range, *IOUS* intraoperative ultrasound, *M0* no distant metastasis, *NAC* neoadjuvant chemotherapy, *NAST* neoadjuvant systemic therapy, *NAT* neoadjuvant therapy, *NR* not reported, *NST* neoadjuvant systemic therapy, *SD* standard deviation, *SLNB* sentinel lymph node biopsy, *TAD* targeted axillary dissection, *Tc-99m* technetium-99m, *ycN* post-neoadjuvant therapy clinical nodal stage.

### Quality assessment

**Fig. 2:**
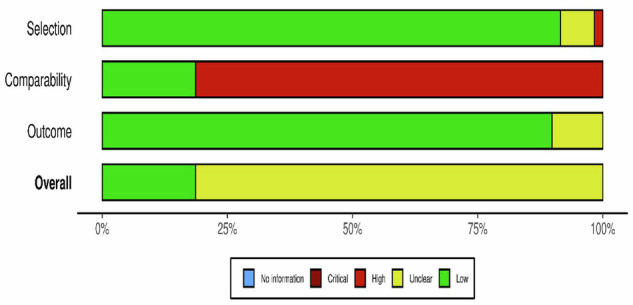
Risk of bias graph items presented as percentages across all included studies. Risk of bias graph presents items as percentages across all included studies. This bar graph visualizes the methodological quality assessment, showing proportions of studies rated as low, high, or unclear risk for domains such as patient selection, index test, reference standard, and flow/timing.

Quality assessment was conducted using the Newcastle-Ottawa Scale (NOS). The overall risk of bias summary is presented in Fig. 2. In the selection domain, the risk of bias was predominantly low (green), with most studies meeting the criteria for high quality in this domain. Comparability domain presented the most significant source of bias within the included studies. Most of the studies were classified as having a high risk of bias (red) regarding comparability, likely due to a lack of control for confounding factors in the study design or analysis. The outcome domain assessment showed that the majority of studies were categorized as low risk (green).

### Synthesis of findings

#### Identification rate

**Fig. 3:**
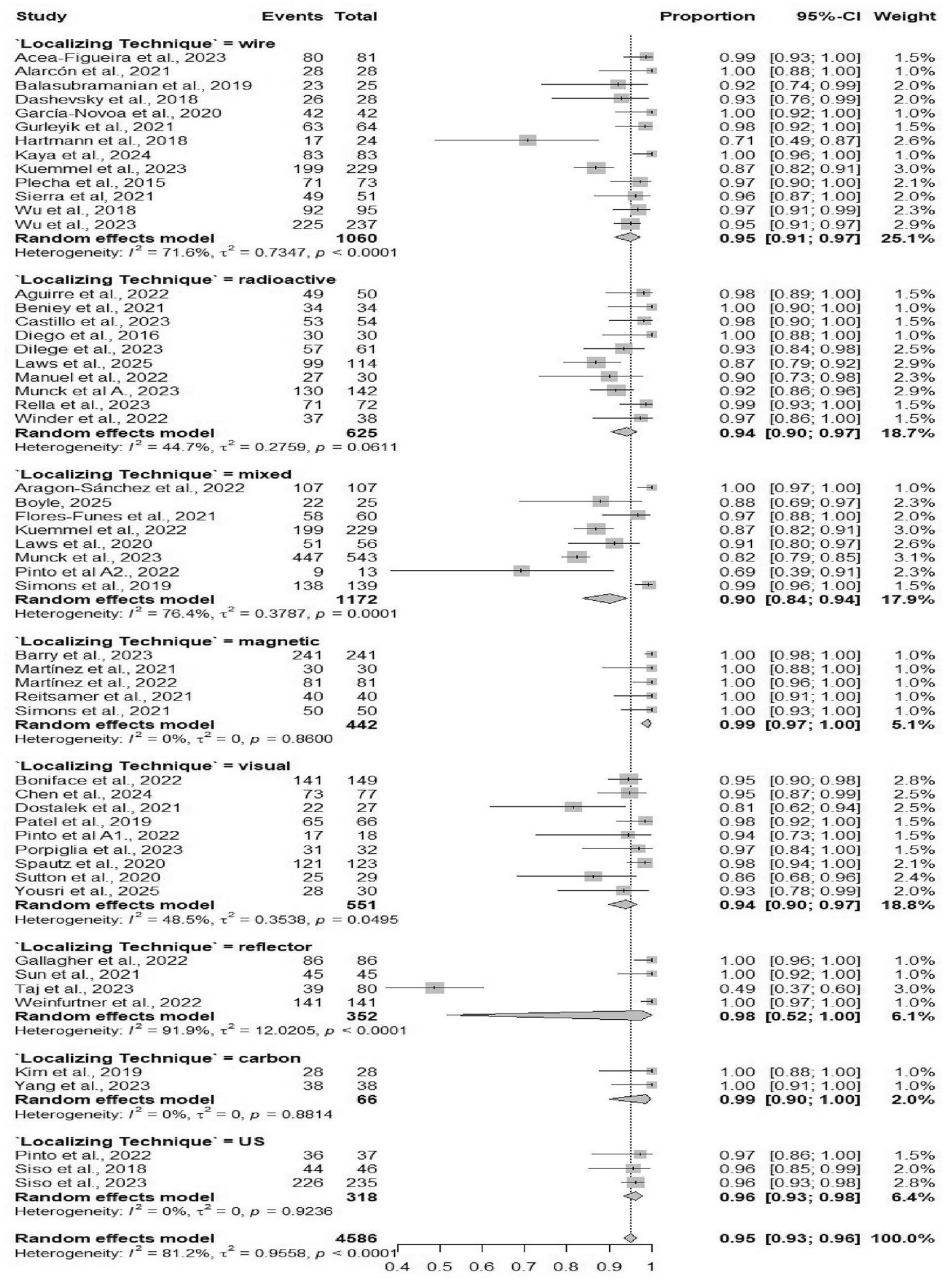
Forest plot showing subgroup analysis of the detection rates by localization techniques. Forest plot displays subgroup analysis of detection rates by localization techniques. Each subgroup (e.g., wire-guided, radioactive seed, magnetic marker) shows individual study effect sizes with 95% confidence intervals, horizontal lines for CIs, squares for point estimates (weighted by sample size), and a diamond for pooled estimate. Vertical line at no-effect (typically 1 for rates), with heterogeneity statistics (*I*², *τ*²) and test for subgroup differences.

**Fig. 4:**
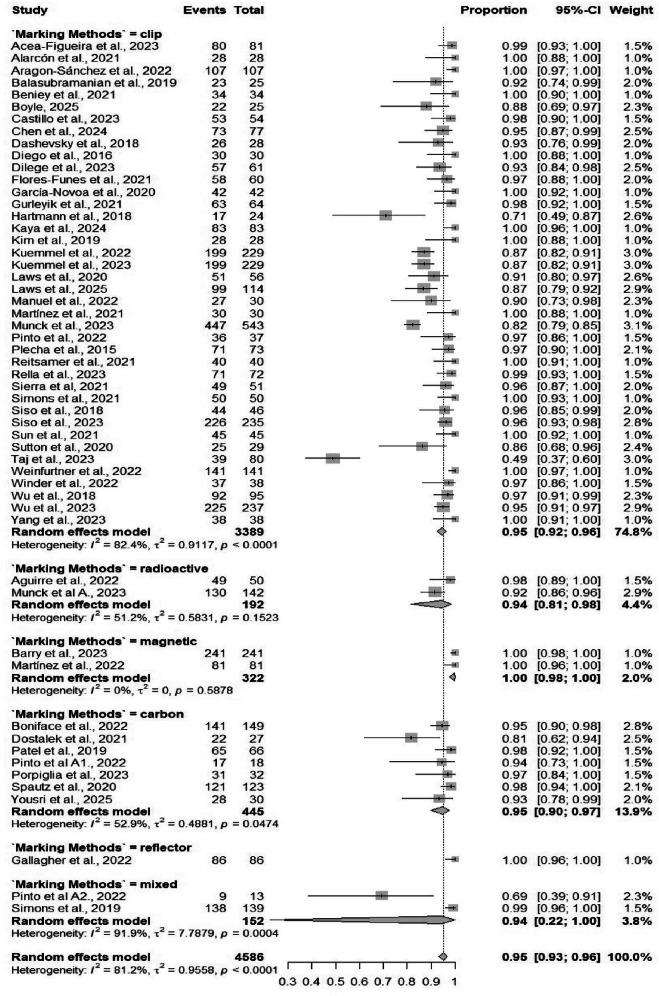
Forest plot showing subgroup analysis of the detection rates by marking methods. Forest plot illustrates subgroup analysis of detection rates by marking methods. Subgroups differentiate methods like hooked-wire, iodine seed, radiofrequency, or tattooing, with study-specific detection rates (e.g., proportion of successful localizations), CIs, pooled diamonds per subgroup, and overall summary. Includes weights, heterogeneity measures, and *p*-values for differences between marking approaches, highlighting optimal methods for surgical precision in breast cancer procedures.

Fifty-four studies comprising 4586 procedures reported data on the identification rate of TAD. The pooled identification rate was 95.1% (95% CI: 93.2%–96.5%) (Table 2), with heterogeneity observed (*I*² = 81.2%) (Fig. S1). Subgroup analyses demonstrated no significant differences when comparing different localizing techniques, and similarly, no significant differences when comparing different marking methods (Figs. 3 and 4). Subgroup analysis based on localizing techniques demonstrated consistently high identification rates across all methods, including magnetic (99%), carbon (98%), and wire-guided (95%), with a statistically significant difference between subgroups (*p* = 0.001). Subgroup analysis by marking methods showed high identification rates for all methods, such as clip-based (94.7%) and carbon (94.9%), without significant between-group differences (*p* = 0.084).

**Table 2:** Pooled estimate of the primary outcomes

Outcome	No. of studies	Total sample size	Pooled estimate (95% CI)	*I*² (%)
Identification rate	54	4586	95.1% (93.2–96.5)	81.2
False-negative rate (FNR)	38	1291	6.37% (5.02–8.04)	0.0
Concordance	45	3293	73.34% (69.58–76.19)	76.5
Overall accuracy	28	1499	94.68% (91.70–96.63)	70.1

Meta-regression analyses showed no significant association between identification rate and either sample size (*p* = 0.327) or mean age (*p* = 0.490). Publication bias was detected by funnel plot examination and Egger’s test (*p* < 0.0001), but it was not significant according to Begg’s test (*p* = 0.0758) (Fig. S5). The sensitivity analysis shows that the pooled identification rate is highly robust. Removing any single study yielded a pooled proportion that remained consistent at approximately 95% (Fig. S9).

#### False-negative rate (FNR)

Thirty-eight studies involving 1291 patients reported FNR outcomes. The pooled FNR was 6.37% (95% CI: 5.02%–8.04%) (Table 2), with no heterogeneity observed (*I*² = 0.0%) (Fig. S2).

**Fig. 5:**
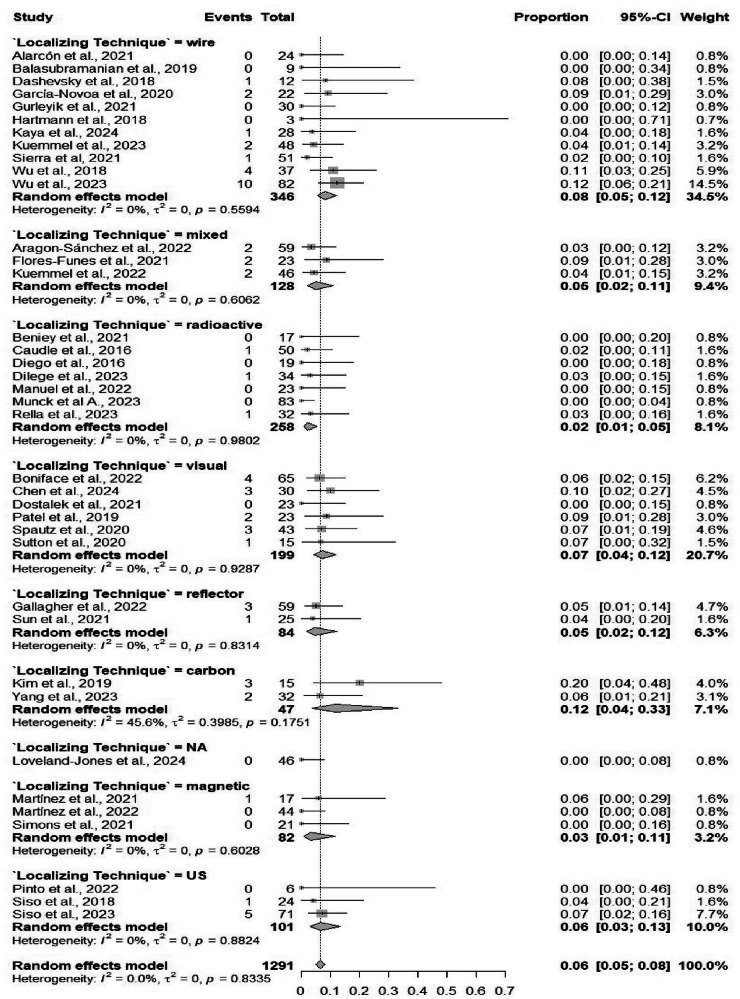
Forest plot showing subgroup analysis of the false negative rates by localizing techniques. Forest plot shows subgroup analysis of false negative rates by localizing techniques. False negatives (missed lesions) are plotted by technique, with effect sizes as proportions or odds ratios, CIs, pooled estimates, and subgroup comparisons. Low false negative rates indicate superior techniques; forest plot reveals variability, heterogeneity, and statistical significance across methods like ultrasound-guided vs. others.

**Fig. 6:**
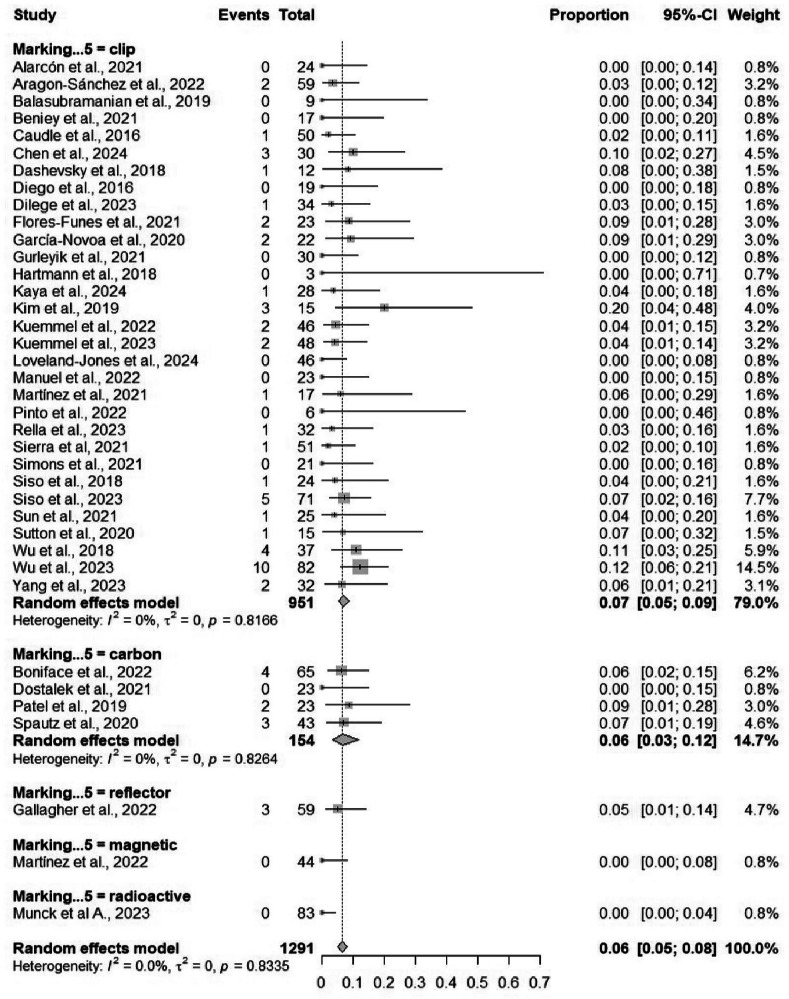
Forest plot showing subgroup analysis of the false negative rates by marking methods. Forest plot depicts subgroup analysis of false negative rates by marking methods. Subgroups compare methods’ failure rates in lesion localization, using inverse variance random-effects pooling, diamond summaries, and tests for differences. Emphasizes methods minimizing surgical re-excisions in oncologic surgery.

Subgroup analyses based on marking methods revealed comparable FNRs across marking methods, including clip-based (6.7%) and carbon (6.5%), with no statistically significant subgroup differences (*p* = 0.303). Similarly, FNRs did not differ significantly across localizing techniques (*p* = 0.144) (Figs. 5 and 6). Meta-regression showed no significant association with age (*p* = 0.947) or sample size (*p* = 0.807). Both Egger’s test (*p* < 0.0001) and Begg’s test (*p* = 0.0429) indicated significant publication bias (Fig. S6). The sensitivity analysis results were highly stable. The pooled proportion remained at 6.4%, and heterogeneity remained at 0%, indicating that no single study skewed the results (Fig. S10).

#### Concordance rate

**Fig. 7:**
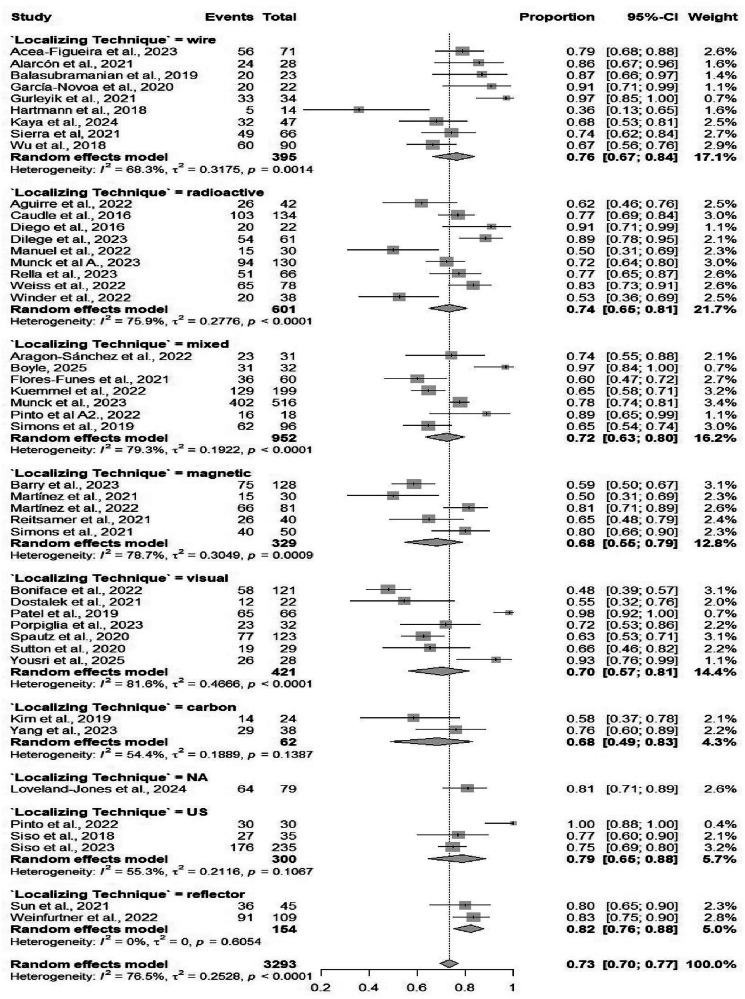
Forest plot showing subgroup analysis of the concordance rates by localizing techniques. Forest plot presents subgroup analysis of concordance rates by localizing techniques. Concordance (agreement between pre- and intraoperative findings) shown per study, pooled by technique, with CIs, heterogeneity, and subgroup tests. High concordance supports technique reliability.

**Fig. 8:**
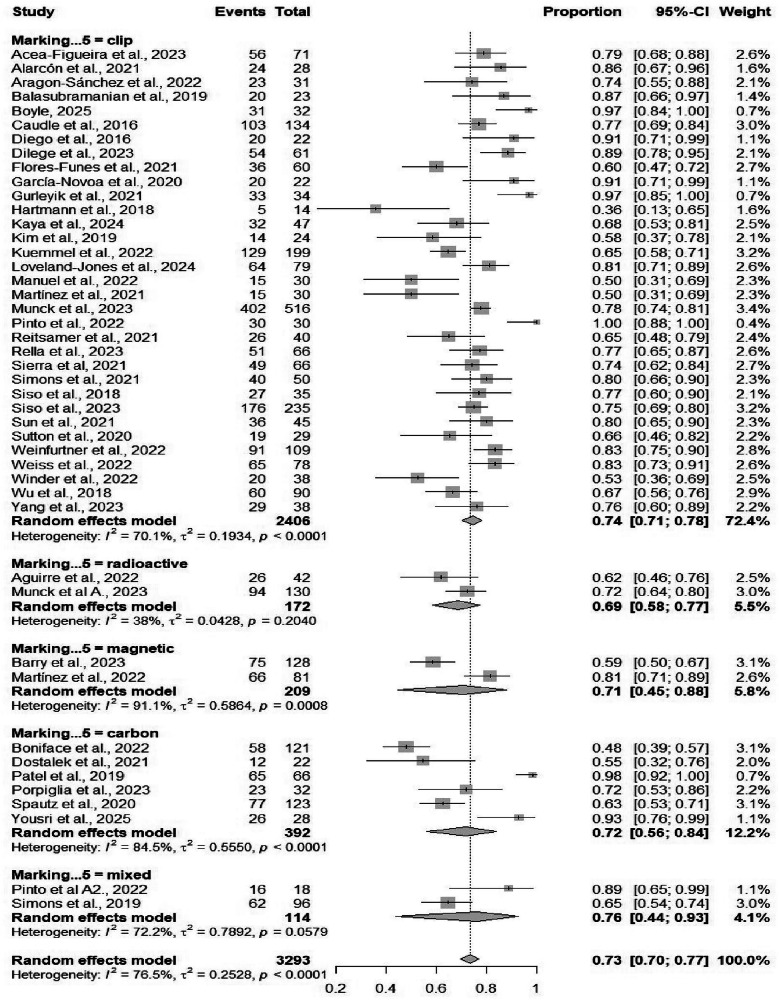
Forest plot showing subgroup analysis of the concordance rates by marking methods. Forest plot illustrates subgroup analysis of concordance rates by marking methods. Displays pooled concordance proportions, aiding selection of methods enhancing surgical accuracy.

Forty-five studies, including 3293 observations, reported concordance. The pooled concordance rate was 73.34% (95% CI: 69.58%–76.79%) (Table 2). High heterogeneity was present (*I*² = 76.5%) (Fig. S3). Subgroup analyses showed similar concordance rates across marking methods, including clip-based (74%), magnetic (71%), and carbon marking (72%), with no significant subgroup differences (*p* = 0.839). Concordance rates also remained comparable across different localizing techniques, with no significant difference (*p* = 0.306) (Figs. 7 and 8).

Meta-regression analyses indicated no statistically significant association between concordance and mean age (*p* = 0.324) or sample size (*p* = 0.905). Publication bias was statistically significant via funnel plot, along with Egger’s test (*p* = 0.045) and Begg’s test (*p* = 0.019) (Fig. S7). The sensitivity analysis showed minimal fluctuation when individual studies were omitted (Fig. S11).

#### Accuracy of nodal staging

**Fig. 9:**
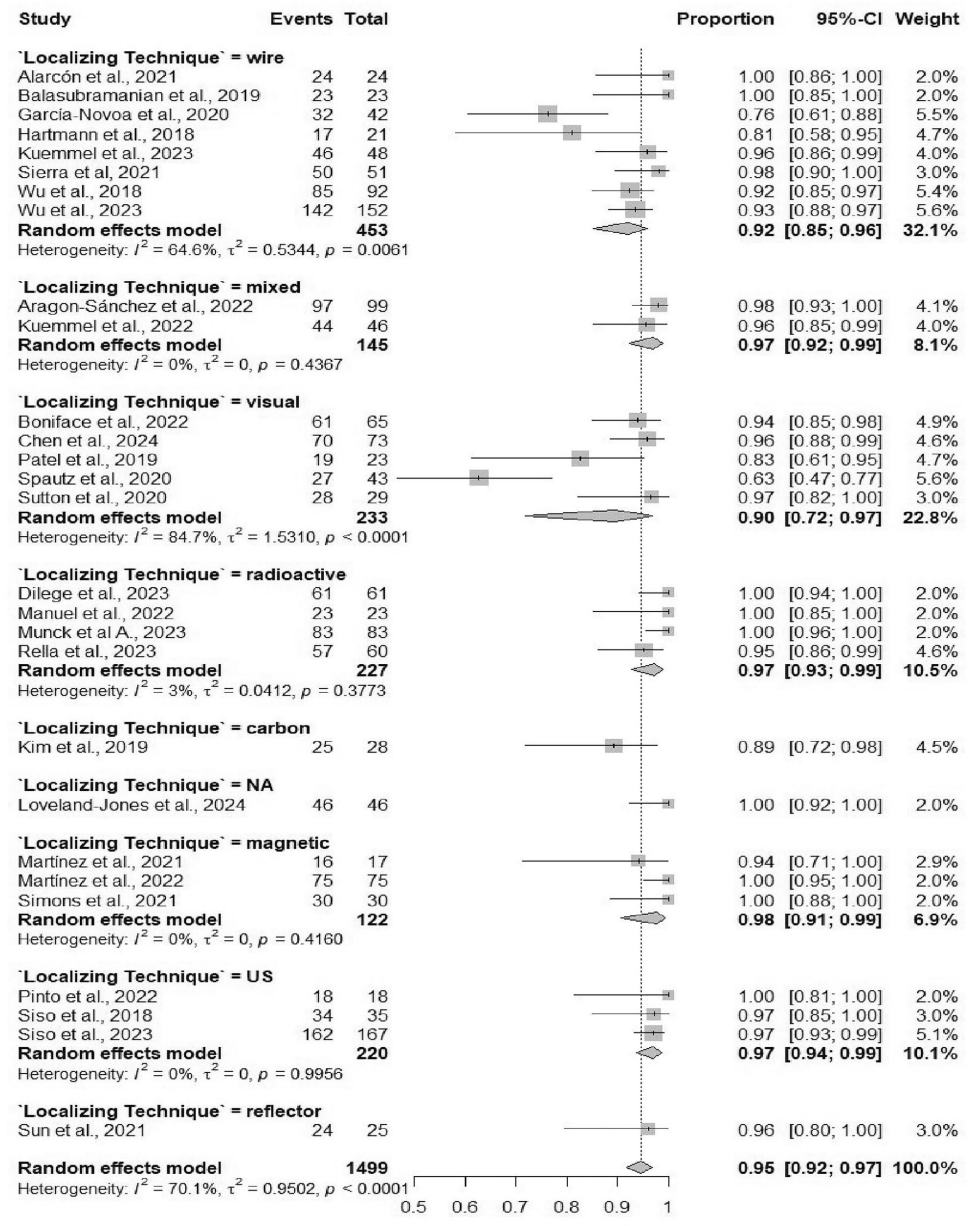
Forest plot showing subgroup analysis of the nodal staging accuracy rates by localizing techniques. Forest plot shows subgroup analysis of nodal staging accuracy rates by localizing techniques. Accuracy in pN staging (e.g., sensitivity for node positivity) pooled per technique, with forest elements revealing performance differences.

**Fig. 10:**
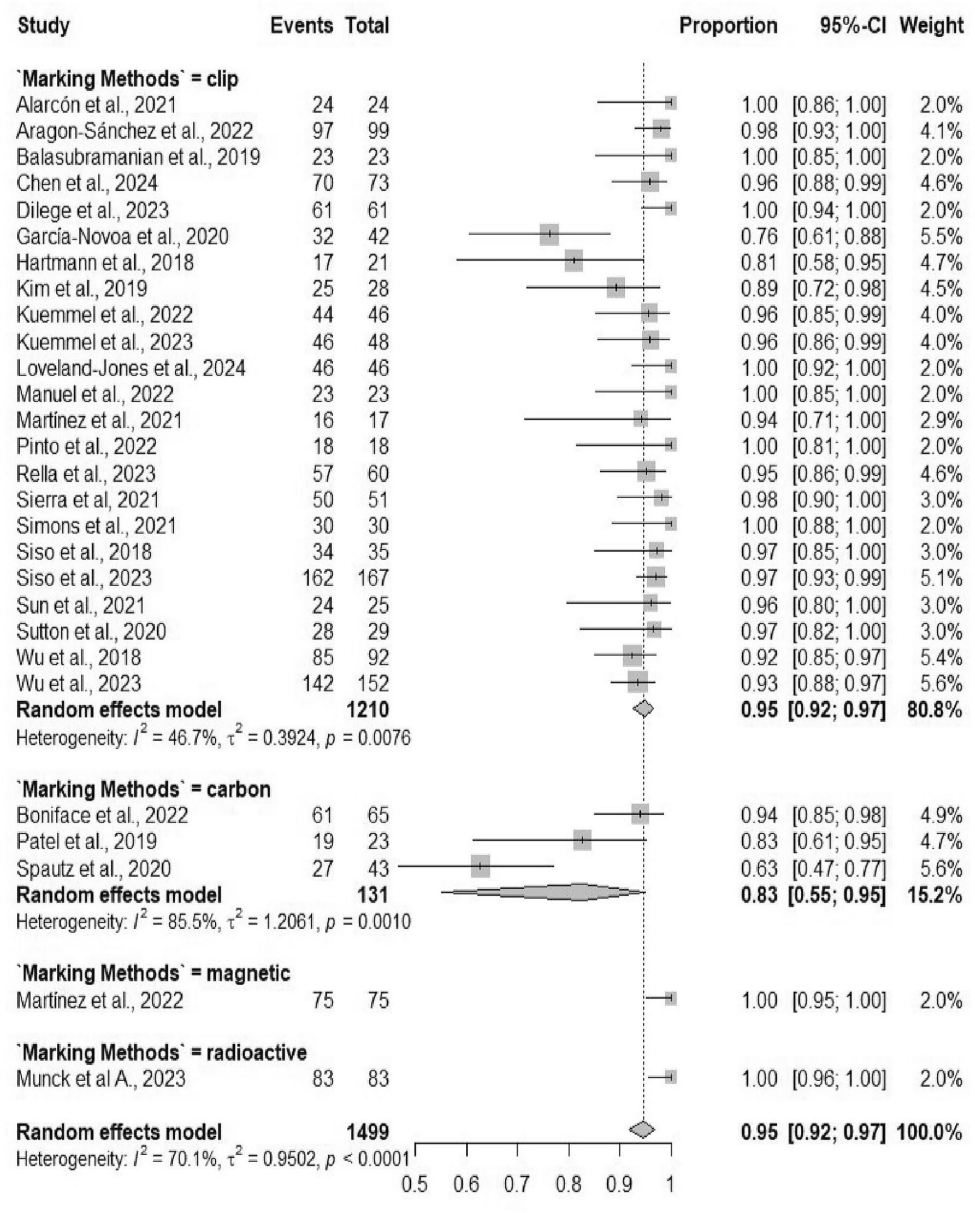
Forest plot showing subgroup analysis of the nodal staging accuracy rates by marking methods. Forest plot displays subgroup analysis of nodal staging accuracy rates by marking methods. Compares marking impacts on staging precision, informing clinical guidelines.

Twenty-eight studies, including 1499 patients, reported overall diagnostic accuracy. The pooled accuracy of TAD was 94.68% (95% CI: 91.70%–96.63%) (Table 2), with moderate heterogeneity (*I*² = 70.1%) (Fig. S4). While there was a difference between different marking methods (*p* = 0.037), subgroup analysis by localizing techniques did not differ significantly (*p* = 0.139) (Figs. 9 and 10).

Meta-regression analyses found no significant association between accuracy and mean age (*p* = 0.490) or sample size (*p* = 0.801). Egger’s test suggested significant publication bias (*p* = 0.0001), whereas Begg’s test was not significant (*p* = 0.072) (Fig. S8). The sensitivity analysis indicated that the results were stable, with minimal fluctuation. However, regarding heterogeneity, the removal of the study by Spautz et al. study caused a drop in I2 to 49.1%, indicating this specific study was a significant source of statistical heterogeneity for the accuracy outcome (Fig. S12).

## Discussion

The current meta-analysis shows that TAD achieves satisfactory results in node identification and produces minimal FNRs while maintaining strong diagnostic performance. The application of this method receives backing from Swarnkar et al., who demonstrated its effectiveness as a dependable, less invasive axillary staging approach^30^. The pooled identification rate of TAD was 95.1%, indicating that the marked node and sentinel nodes can be successfully retrieved in the vast majority of procedures across diverse marking and localization techniques^29^. The pooled false‑negative rate (FNR) of 6.37% and overall accuracy of 94.68% are within widely accepted thresholds for oncologic safety, with concordance between targeted and sentinel nodes of 73.34%, suggesting that TAD can reliably reflect the true axillary status after NST^30^. The research results demonstrate that TAD functions effectively as an alternative to ALND for patients who began with clinical node positivity and achieved negative node status after NST treatment^31^.

The current estimates have been confirmed by previous pooled analyses, which showed TAD identification rates that matched or were slightly lower, and FNRs that remained low^29,30^. An early study of 13 TAD patient groups, which included 521 patients, revealed a 5.2% FNR, which closely matches the present pooled FNR of 6.37%. Thus, demonstrating that TAD performance remains stable across different time periods and surgical methods^30^. The SenTa study by Kuemmel et al demonstrated that TAD was successful in 199 patients who achieved superior 3-year invasive disease-free survival after ALND was avoided in patients who showed a response to treatment selection^31^. This indicates that the diagnostic performance observed in the current meta‑analysis translates into favorable oncologic outcomes.

The current identification rate of 95.1% matches or stands at a slightly lower level than the near-universal retrieval rates of radioactive seed-guided TAD, reported in the literature, where pooled localization and retrieval rates reached 97–100%^32^. The marking technique-focused overviews demonstrate identification rates that exceed 95% and FNR values that stay below 9% when testing various systems, including clips, seeds, carbon tattoo, magnetic, and radar-based systems^33^. The subgroup analyses from this meta-analysis demonstrate that all techniques perform at about the same level of effectiveness. A recent systematic review on TAD marker type and timing found wide variation in imaging identification but consistently high intraoperative retrieval, again compatible with the high pooled identification rate observed here^29^.

Compared with SLNB alone after NST in initially node‑positive disease, the present FNR for TAD is substantially lower. The previous meta-analyses, which studied SLNB alone in this context, found false negative rates between 13–17%, which has created concerns about understaging and supports the development of targeted methods^30,34^. Prospective trials (ACOSOG Z1071, SENTINA, SN-FNAC, GANEA 2) confirmed high overall SLNB FNRs (8.6–18.2%) post-NAC in cN+ disease, but optimized protocols—retrieval of ≥2–3 SLNs with dual-agent mapping—yielded FNRs <10%, comparable to TAD irrespective of marked node inclusion^22–25^. These diagnostic findings translate to equivalent long-term outcomes: in a multicenter cohort by Montagna et al.^35^, 5-year axillary recurrence rates were comparable between optimized SLNB (dual mapping, ≥3 SLNs; 0.8% at 3 years) and TAD (0.5%; *P* = 0.55), with low locoregional (2.7%) and invasive recurrence overall, supporting omission of ALND without mandatory marked node retrieval. Our TAD meta-analysis FNR (6.37%) reinforces this equivalence, endorsing either approach for nodal pCR confirmation when technical criteria are met, thus broadening de-escalation options beyond resource-intensive marking. The TAD methods, which combine clipped node removal with SLNB, have demonstrated false negative rates below 10% through various single-method studies, including the TATTOO trial, which used carbon tattooing, and the guidewire and seed-based TAD methods that achieved false negative rates between 4 and 7%^36–38^. This matches the current pooled estimate.

De-escalated axillary surgery through TAD methods has been shown to be safe based on data that tracks patient results over extended periods. A meta-analysis study analyzed multiple studies about SLNB alone and MARI/TAD in node-positive patients who achieved nodal pCR, found that axillary recurrence rates stayed between 1.5 and 2.1% with no evidence that ALND would improve patient outcomes in these cases^39^. The analysis of registry data demonstrates that SLNB, TAD, and ALND produce equivalent invasive disease-free survival results when adjusted for treatment response and tumor characteristics^31,40^. This proves that TAD’s low FNR value is oncologically acceptable. Nevertheless, some authors dispute whether SLNB functions as a standalone method for patients who show excellent response to treatment because various studies demonstrate axillary recurrence rates below 2%, which creates uncertainty about the necessity for all patients who convert to ycN0 status^10,41^.

Such a high identification rate with correspondingly low FNRs could be achieved because of a combination of pre-treatment pathological confirmation, accurate marker placement, and dual-component surgery (marked node plus sentinel nodes). In marking the biopsy-proven positive node pre-NST, one is ensuring that the initially involved axillary basin is properly interrogated after systemic therapy, which would address the principal limitation of SLNB alone, namely, the sampling of a potentially different nodal drainage pathway that may subsequently miss residual microscopic disease^30,33^. The extraction of the clipped or tattooed node in conjunction with SLNB enhances the possibility of sampling the residual diseased compartment, thus leading to the reduced FNR observed across modern TAD cohorts^35,38^.

From a biological point of view, a high nodal pCR attained after modern NST, especially in the HER2-positive and triple-negative subtypes, strongly favors the option of axillary de-escalation^31,35^. In these patients, systemic therapy eliminates nodal metastases in a significant number, and therefore, the vast majority of ALNDs turn out to be positive for only negative nodes and carry significant morbidity for the patients. TAD utilizes this biology and stresses the surgical approach on nodal verification rather than on complete clearance, thereby matching local treatment intensity to systemic response^31,35^. Differences in concordance between the targeted node and SLNs may vary across studies, likely due to differences in lymphatic remodeling after NST, tumor biology, and mapping technique, which may partially explain the moderately low concordance rate (~73%) found in this meta‑analysis, despite the overall high accuracy^33^.

The findings at present are in strong agreement with former systematic reviews focused on TAD and huge prospective cohorts, which again point out that identification rates are high with FNRs below 10%^29,30^. For instance, recent series data using radioactive iodine seeds, magnetic seeds, or carbon tattoos show detection rates ranging from 94 to 100% and FNRs from 4 to 7%, in close agreement with pooled estimates described here^32,36,37,42^. Further, international surveys of practices reveal that TAD rapidly became the preferred axillary route for concerned cN1 patients converted to ycN0, which resonates with the good diagnostic summary of this meta-analysis^43^.

However, some inconsistencies are notable. First, the pooled concordance rate of targeted and sentinel nodes for this analysis (73.34%) seems somewhat higher than the 58–60% reported within some unique single‑center TAD series, particularly in those using seed or magnetic-guided techniques^32–35,38,39,43,44^. This difference may arise from the inclusion of studies that impose strict mapping protocols accompanied by high sentinel node retrieval numbers or possibly the greater differences in imaging and surgical expertise that enhance the overlap between drainage pathways^33^. Secondly, while marking and localization methods appear to perform similarly in this meta-analysis, individual studies suggest technique-specific advantages, like the logistical convenience and high retrieval rates of iodine seeds or the regulatory simplicity of magnetic and carbon techniques^29,32,33^. The lack of statistically significant differences in most subgroup analyses in this study might be due to insufficient power, residual confounding, and heterogeneity in operator experience, rather than genuine equivalence between each technique tested^29^.

Another area of contention is whether TAD is clinically necessary or whether SLNB alone is adequate. While registry and retrospective comparative studies from high-volume centers suggest that SLNB alone and TAD provide similar rates of regional control in selected responders, some experts still caution that an FNR of about 13–17% with SLNB alone in historic series has been unacceptably high and that TAD provides an added safety margin^10,34,41^. The presently pooled FNR of 6.37% supports that view but does not definitely answer whether TAD should be a standard consideration in all cN+ responders, especially in the light of emerging outcome data showing low recurrence when either method is combined with optimized systemic therapy and radiotherapy^39,40^.

The high accuracy and low FNRs of TAD mapped out in this meta-analysis mainly have implications for axillary management guidelines in node-positive breast cancer patients who have undergone NST. In fact, TAD is a reliable way to identify nodal pCR and safely omit ALND in a large proportion of patients, thus preventing the development of lymphedema, shoulder dysfunction, and neuropathic pain after extensive nodal clearance^31,35^. Real-life data collected from the SenTa registry as well as other cohorts signal that there proves very minimal axillary recurrence from TAD alone performed on ypN0 carefully selected patients, with survival outcomes maintained, further supporting the clinical safety of de-escalation constructs directed by precise axillary staging^31,40^.

For one, evidence of consistent performance across multiple marking and localization techniques indicates that centers may start TAD using localized technologies like clips with wire localization, radioactive seeds, carbon tattooing, or magnetic seeds, without even jeopardizing the reliability of the diagnosis, as long as the targeted node is well defined and retrieved^33^. This has public health meaning, as it proposes that TAD can be employed for resource-poor settings: carbon tattooing produced FNRs that are comparable to the more sophisticated radioactive alternatives, which is very attractive indeed in regions where there are regulatory or logistical obstacles to the use of radioactive seed^29,36,45^. Finally, less extensive ALND could be avoided for a large percentage of women with node-positive disease using TAD-guided approaches, thus improving the quality of life and functional outcomes at a population level while still preserving oncologic safety, an increasingly major objective in modern breast cancer care^39^.

In particular, prospective cohort studies (most studies), retrospective cohorts were used, and registries were examined from differing geographic locations (mostly Europe, USA, China) and practice settings widely ranging from high-volume specialist centers to general hospital breast programs. This wide variability introduces differences in criteria for patient selection (e.g., nodal staging approval) in the long run and reporting of outcomes. While heterogeneity was substantial for the identification rate (*I*² = 81.2%) and concordance (*I*² = 76.5%), the estimated value of zero heterogeneity calculated for the FNR proposes that the efforts to ascertain this important safety measure congregated around a clinically meaningful estimate. Possibly, in some studies, full completion of axillary dissection was not done for all patients with negative TAD, thus potentially underestimating the FNR if residual axillary disease was undetected. On the other hand, other studies performed ALND selectively, creating a bias in FNR estimates if ALND was more likely to be performed in cases with clinical suspicion for residual disease. Ideally, all patients included in TAD studies should undergo ALND to allow unbiased FNR computation, but such study designs are increasingly considered to raise ethical concerns, given the emerging evidence for TAD safety.

While TAD is supported by the literature due to its lower FNR, it is not universally available. But for sure, its substitute is not ALND in the era of treatment de-escalation. Dual-tracer SLNB remains an acceptable alternative for axillary staging, following evidence from trials such as ACOSOG Z1071 and the SENTINA Trial that recommend specific technical criteria to lower the fNR, including the use of dual tracers and the retrieval of at least three sentinel lymph nodes^24,25^. In addition, growing evidence over the last few years indicates that, despite the known FNR of single-tracer classic SLNB in the post-neoadjuvant setting, it remains a valid option in axillary management without affecting regional recurrence rates or overall survival^10,46–49^. However, we acknowledge that the absence of TAD may still be associated with a relatively higher FNR. While there is comparison in the meta-analysis of marking technique performances, timing of marker placement (pre-NAC versus post-NAC), number of nodes marked with disease status N2, and marker placement-to-surgery duration introduce variability. It indeed marks confirmed metastatic disease at the pre-treatment condition, but has evolved more toward a pre-NAC wire-free technology placement since the period of this study (2016–2025). Many studies have 3–5 year outcomes; the longest follow-up available is about 5 years^31, 50^. Longer follow-up data (7–10 years) will give the most confidence in maintaining the durability of TAD-based de-escalation strategies with regard to late locoregional recurrence patterns. Around 70% of studies were from Europe and North America that contributed to the study population; contributions from China and a few other places were limited. Thus, selection bias on patient demographic characteristics (42–60 years of mean age), tumor biology, and access to advanced imaging and localization technologies may come in. How applicable these findings will be to populations with different age distributions, tumor burdens, or resource constraints should also be considered. A direct comparison between one-step and two-step TAD approaches was not feasible due to inconsistent reporting across studies, representing a limitation, especially given emerging evidence suggesting potential advantages of single-device (one-step) strategies. Future studies comparing outcomes of dual-technique SLNB versus TAD in resource-limited settings are warranted.

The meta-analysis concludes that targeted axillary dissection is an extremely reproducible and accurate technique for staging axillae in women with initially node-positive breast cancer only undergoing neoadjuvant systemic therapy and achieving clinical nodal downstaging. In pooled identification rates, the elements are 95.1%, FNRs: 6.37%, and diagnostic accuracy: 94.68%, all of which establish TAD as superior to SLNB alone and support its acceptance as a standard surgical approach in patients selected according to the evidence strength. There is no heterogeneity degree in FNR estimates, and results are consistent across different marking techniques and localization methods; thus, solid and generalizable findings can be drawn from these results. Emerging data demonstrating the oncologic safety of TAD without completion ALND in those patients with ypN0, along with having retrieved three or more nodes, provide a scientific basis for further de-escalation of axillary surgery, with reduced morbidity from treatment but maintaining oncologic efficacy. Future prospective randomized trials and consensus guidelines will be necessary to standardize TAD protocols, define the optimal patient selection criteria, and define TAD as the de facto standard for axillary staging in this important clinical circumstance, regarding oncologic outcomes and quality of life for patients in active management for breast cancer with neoadjuvant systemic therapy.

## Methods

### Protocol registration

This systematic review, along with meta-analysis, was registered under the International Prospective Register of Systematic Reviews (PROSPERO), with the number CRD420250651586. Compliance with the Cochrane Handbook for Systematic Reviews of Interventions, for systematic review and meta-analysis per Optimal reporting of systematic reviews and meta-analyses, was undertaken as above.

### Search strategy

These parameters developed into a search strategy according to PICO: population, intervention, comparison, and outcome. The subsequent question is: In clinically node-negative adult breast cancer patients with initial node-positive disease treated with neoadjuvant systemic therapy, what is the diagnostic performance of TAD in terms of identification rate, false negative rates, concordance rate, and nodal staging accuracy, and how do different marking methods and localizing techniques influence these outcomes? Systematic review design in English from inception to April 2025 was followed in the databases PubMed, Scopus, Cochrane Library, and Web of Science. There were no limits on publication dates and geographical location for any references. Controlled vocabulary and keywords were used in searching. The detailed search strategy and specific keywords are addressed in the supplementary file. Other reference lists were reviewed for possible relevance, while bibliographies of included studies were screened for articles that may have been missed.

### Eligibility criteria

Adult breast cancer patients (≥18 years) diagnosed with node-positive disease and treated with neoadjuvant therapy who achieved clinically negative node status confirmed either by imaging or clinical examination at an eligible study will qualify for inclusion in this review. Randomized controlled trials, cohort studies, case-control studies, or retrospective analyses that provide individual-level data on the performance of TAD will be eligible study designs. The required intervention will include standard TAD methodology that combines the removal of pre-marked or pre-clipped axillary nodes using validated localization methods with the performance of SLNB. In the TAD cohorts included in this review, a completion ALND will serve as a reference standard for validating the FNR and/or accuracy of TAD using standard diagnostic methods.

Studies were excluded if they involved pediatric or non-human populations, case reports, conference abstracts, narrative reviews, opinion pieces, or methodologically flawed/overlapping datasets. Non-English full-text articles, grey literature, preprints, metastatic or recurrent breast cancer cases, or patients who did not achieve clinical node-negative status post-neoadjuvant therapy were excluded. Studies employing non-TAD interventions, non-standard TAD methodology (e.g., node removal without prior marking), or studies focused on non-axillary management (e.g., imaging or systemic therapy trials without nodal evaluation) or outdated techniques no longer considered standard care.

### Outcome definition

The main outcome measures of this study included the accuracy of nodal staging, the identification rate, the concordance rate, and the FNR during TAD in breast cancer patients. Secondly, to assess the effects of different marking and localizing techniques on TAD outcomes. The identification rate of TAD was defined as the proportion of procedures with successful intraoperative retrieval of both the pre-marked target node (clip/marked) and sentinel lymph node(s) (SLNs). FNR was defined as the proportion of patients who had residual axillary nodal disease on completion of ALND, but in whom TAD was negative. Accuracy of nodal staging was defined as the proportion of patients in whom TAD correctly classified the final axillary nodal status compared with the reference standard, which was completion of ALND when available. Therefore, accuracy was calculated in studies where ALND was performed after TAD, allowing direct verification of true-positive and true-negative cases. Concordance rate was defined as the proportion of cases in which the previously clipped (biopsy-proven positive) lymph node is identified as one of the SLNs during surgery.

### Study selection

EndNote 21 was used to eliminate duplicate studies; then two independent reviewers screened titles and abstracts before systemically applying inclusion and exclusion criteria, with divergence resolved by consensus with a third reviewer. The following data were extracted and reported by two independent authors: surname of the first author; year and location where the study was conducted; funding source; conflict of interest; sample size; description of the population; age; tumor type; initial nodal status; type of neoadjuvant therapy; clinical node-negative criteria; type of intervention; type of marking technique; type of localization technique; total number of axillary LNs harvested; method of SLNB; identification rate of the targeted nodes; false negative rate; accuracy of nodal staging; concordance rate; survival outcomes; postoperative complications; and length of follow-up. Data were extracted from text, tables, and figures. The variables under question were unclear from the presentation. Data were then double-checked by a third independent reviewer for accuracy, consistency, and comprehensiveness.

### Quality assessment and publication bias

In evaluating the quality of nonrandomized studies, the NOS criteria were used. Hence, there must be more than one question on selection, comparability, and outcome domains. The methodological quality of the included studies was independently assessed by two reviewers. Discrepancies between reviewers were resolved through discussion and, when necessary, consultation with a third reviewer. Each study could receive a maximum of nine stars, with higher scores indicating better methodological quality. Studies scoring 7–9 stars were considered high quality, 4–6 stars moderate quality, and ≤3 stars low quality. The quality assessment results were used to inform the interpretation of the findings, but did not serve as exclusion criteria for study selection. The funnel plot and Begg’s test were employed to evaluate publication bias.

### Statistical analysis

All quantitative analyses were performed using R software (version 4.4.3) with the Meta packages. The research determined single-arm proportions for outcome analysis, which included identification rate, FNR, and concordance and overall accuracy metrics. The random-effects model with the DerSimonian–Laird method and logit transformation was used to combine proportions for variance stabilization. The *I*² statistic served to evaluate between-study heterogeneity, with values 25%, 50%, and 75% indicating low, moderate, and high heterogeneity, respectively. The study performed subgroup analyses, which followed the pre-established plan to evaluate different localization techniques and marking methods by applying the Q test for statistical differences between groups. Random-effects meta-regression was performed for relevant continuous covariates when at least 10 studies per variable were available. The study used leave-one-out analysis together with influence diagnostic tests to assess the stability of its results. Publication bias was evaluated using funnel plots, Egger’s regression test, and Begg’s rank correlation test. The study used two-sided tests with a 0.05 significance threshold to present results as pooled proportions with 95% confidence intervals. Forest plots were generated for each outcome, and subgroup/meta-regression outputs were reported in the main text and Supplementary as appropriate.

## Supplementary information

Supplementary Information

## Data Availability

All data generated or analyzed during this study are included in this published article and its supplementary information files.

## Code availability

The statistical analyses were performed using R software. The code used for the analyses is available from the corresponding author upon reasonable request.
