# Supplementary material for: Targeted axillary dissection in breast cancer patients: a systematic review and meta-analysis
Source: NPJ Breast Cancer. 2026 Jun 5;12:79. doi: 10.1038/s41523-026-00984-3 (PMC13241489; doi:10.1038/s41523-026-00984-3)
Supplement: Supplementary file 1 — Supplementary Information [file 41523_2026_984_MOESM1_ESM.pdf]

Targeted axillary dissection in breast cancer patients: A systematic review and meta-analysis

Mohammed. N. Abdelaziz[1*], Sagad O.O. Mohamed[2+], Mohammed Hesham Nagi[1+], Ibrahim Saleh Alawadi[1], Youstina Mohsen[1], Sohaila Essam Ibrahim[3], Radwa M. Abdelsattar[1], Asmaa. N. Abdelaziz[1], Mohamed Yasser[1], Sherif Wael[1], Hend Ahmed[1], Magdy Shehab[1], Khaled M Abdelwahab[4], Omar Hamdy[4]

1. Faculty of Medicine, Mansoura University Hospital, Mansoura, Egypt.
2. University of Khartoum, Khartoum, Sudan.
3. kasr alainy, Faculty of medicine, Cairo University Hospital, Cairo, Egypt.
4. Surgical Oncology Department, Oncology Centre, Mansoura University, Mansoura, Egypt

*Correspondence: Mohammed. N. Abdelaziz: Faculty of Medicine, Mansoura University, Egypt. Postal address: Arab Republic of Egypt, Al-Daqahlia Governorate, Mansoura, Mansoura. Postal office number 35516. E-mail: mohammednasser@std.mans.edu.eg ORCID: 0000-0003-2699-2901.

+ Mohammed Hesham Nagi and Sagad O.O. Mohamed have equally contributed.

PICO

Population (P): Breast cancer patients with initially node-positive disease who became clinically node-negative after neoadjuvant therapy.

Intervention (I): Targeted axillary dissection (TAD), which includes sentinel lymph node biopsy (SLNB) combined with the removal of marked nodes.)

Comparison (C): Standard axillary lymph node dissection (ALND) or sentinel lymph node biopsy (SLNB) alone.

Outcomes (O): Accuracy of nodal staging, Detection rate & false negative rate, recurrence rates, survival outcomes, and postoperative complications. Search strategy:

((Breast cancer) OR (mammary cancer) OR (breast malignancy) OR (breast adenocarcinoma) OR (carcinoma of the breast) AND (node-positive OR nodal involvement) OR (positive lymph node) OR (axillary metastasis) OR (metastatic lymph node*)) AND ((node-negative) OR (nodal clearance) OR (negative lymph node*) OR (axillary response) OR (pathologic node-negative)) AND ((neoadjuvant therapy) OR (neoadjuvant chemotherapy) OR (preoperative therapy) OR (induction chemotherapy) OR (primary systemic therapy)) AND ((axillary targeted surgery) OR (selective axillary dissection) OR (marked lymph node removal) OR (clipped node excision) OR (sentinel lymphadenectomy) OR (targeted lymph node biopsy) OR (TAD procedure) OR (dual-tracer technique)) AND ((complete axillary dissection) OR (radical axillary surgery) OR (level I-II dissection) OR (axillary clearance) OR (standalone SLNB) OR (sentinel-only biopsy) OR (conventional axillary surgery)) AND ((lymph node assessment) OR (axillary staging) OR (nodal status accuracy) OR (pathologic staging) OR (sensitivity rate) OR (identification rate) OR (miss rate) OR (diagnostic yield))*

Last date of searching: April 2025

1. PubMed (NCBI)

PubMed relies on **MeSH terms** and field tags like [tiab] (title/abstract) or [MeSH Terms]. This tailored search includes both indexed and free-text terms:

N= 860

From advanced search:**((("breast neoplasms"[MeSH Terms] OR ("breast"[All Fields] AND "neoplasms"[All Fields]) OR "breast neoplasms"[All Fields] OR ("breast"[All Fields] AND "cancer"[All Fields]) OR "breast cancer"[All Fields] OR ("breast neoplasms"[MeSH Terms] OR ("breast"[All Fields] AND "neoplasms"[All Fields]) OR "breast neoplasms"[All Fields] OR ("mammary"[All Fields] AND "cancer"[All Fields]) OR "mammary cancer"[All Fields]) OR (("breast"[MeSH Terms] OR "breast"[All Fields] OR "breasts"[All Fields] OR "breast s"[All Fields]) AND ("malign"[All Fields] OR "malignance"[All Fields] OR "malignances"[All Fields] OR "malignant"[All Fields] OR "malignants"[All Fields] OR "malignities"[All Fields] OR "malignity"[All Fields] OR "malignization"[All Fields] OR "malignized"[All Fields] OR "maligns"[All Fields] OR "neoplasms"[MeSH Terms] OR "neoplasms"[All Fields] OR "malignancies"[All Fields] OR "malignancy"[All Fields])) OR (("breast"[MeSH Terms] OR "breast"[All Fields] OR "breasts"[All Fields] OR "breast s"[All Fields]) AND ("adenocarcinoma"[MeSH Terms] OR "adenocarcinoma"[All Fields] OR "adenocarcinomas"[All Fields] OR "adenocarcinoma s"[All Fields])) OR ("breast neoplasms"[MeSH Terms] OR ("breast"[All Fields] AND "neoplasms"[All Fields]) OR "breast neoplasms"[All Fields] OR ("carcinoma"[All Fields] AND "breast"[All Fields]) OR "carcinoma of the breast"[All Fields])) AND ("node-positive"[All Fields] OR (("nodal"[All Fields] OR "nodally"[All Fields] OR "nodals"[All Fields]) AND ("involve"[All Fields] OR "involved"[All Fields] OR "involvement"[All Fields] OR "involvements"[All Fields] OR "involves"[All Fields] OR "involving"[All Fields] OR "involvment"[All Fields])))) OR (("positive"[All Fields] OR "positively"[All Fields] OR "positiveness"[All Fields] OR "positives"[All Fields] OR "positivities"[All Fields] OR "positivity"[All Fields]) AND ("lymph"[MeSH Terms] OR "lymph"[All Fields] OR "lymphs"[All Fields] OR "lymphe"[All Fields]) AND "node*"[All Fields]) OR (("axilla"[MeSH Terms] OR "axilla"[All Fields] OR "axillary"[All Fields] OR "axillaries"[All Fields] OR "axillaris"[All Fields]) AND ("metastasi"[All Fields] OR "neoplasm metastasis"[MeSH Terms] OR ("neoplasm"[All Fields] AND "metastasis"[All Fields]) OR "neoplasm metastasis"[All Fields] OR "metastasis"[All Fields])) OR (("metastatically"[All Fields] OR "metastatics"[All Fields] OR "metastatization"[All Fields] OR "metastatize"[All Fields] OR "metastatized"[All Fields] OR "metastatizing"[All Fields] OR "secondary"[MeSH Subheading] OR "secondary"[All Fields] OR "metastatic"[All Fields]) AND ("lymph"[MeSH Terms] OR "lymph"[All Fields] OR "lymphs"[All Fields] OR "lymphe"[All Fields]) AND "node*"[All Fields])) AND ("node-negative"[All Fields] OR (("nodal"[All Fields] OR "nodally"[All Fields] OR "nodals"[All Fields]) AND ("clearance"[All Fields] OR "clearances"[All Fields])) OR (("negative"[All Fields] OR "negatively"[All**

Fields] OR "negatives"[All Fields] OR "negativities"[All Fields] OR "negativity"[All Fields]) AND ("lymph"[MeSH Terms] OR "lymph"[All Fields] OR "lymphs"[All Fields] OR "lymphe"[All Fields]) AND "node*"[All Fields]) OR (("axilla"[MeSH Terms] OR "axilla"[All Fields] OR "axillary"[All Fields] OR "axillaries"[All Fields] OR "axillaris"[All Fields]) AND ("response"[All Fields] OR "responses"[All Fields] OR "responsive"[All Fields] OR "responsiveness"[All Fields] OR "responsivenesses"[All Fields] OR "responsives"[All Fields] OR "responsivities"[All Fields] OR "responsivity"[All Fields])) OR (("pathologic"[All Fields] OR "pathologically"[All Fields] OR "pathologics"[All Fields] OR "pathology"[MeSH Terms] OR "pathology"[All Fields] OR "pathological"[All Fields]) AND "node-negative"[All Fields])) AND ("neoadjuvant therapy"[MeSH Terms] OR ("neoadjuvant"[All Fields] AND "therapy"[All Fields]) OR "neoadjuvant therapy"[All Fields] OR ("neoadjuvant therapy"[MeSH Terms] OR ("neoadjuvant"[All Fields] AND "therapy"[All Fields]) OR "neoadjuvant therapy"[All Fields] OR ("neoadjuvant"[All Fields] AND "chemotherapy"[All Fields]) OR "neoadjuvant chemotherapy"[All Fields]) OR ("neoadjuvant therapy"[MeSH Terms] OR ("neoadjuvant"[All Fields] AND "therapy"[All Fields]) OR "neoadjuvant therapy"[All Fields] OR ("preoperative"[All Fields] AND "therapy"[All Fields]) OR "preoperative therapy"[All Fields]) OR ("induction chemotherapy"[MeSH Terms] OR ("induction"[All Fields] AND "chemotherapy"[All Fields]) OR "induction chemotherapy"[All Fields]) OR (("primaries"[All Fields] OR "primary"[All Fields]) AND ("systemic"[All Fields] OR "systemically"[All Fields] OR "systemics"[All Fields]) AND ("therapeutics"[MeSH Terms] OR "therapeutics"[All Fields] OR "therapies"[All Fields] OR "therapy"[MeSH Subheading] OR "therapy"[All Fields] OR "therapy s"[All Fields] OR "therapys"[All Fields]))) AND ((("axilla"[MeSH Terms] OR "axilla"[All Fields] OR "axillary"[All Fields] OR "axillaries"[All Fields] OR "axillaris"[All Fields]) AND ("target"[All Fields] OR "targetability"[All Fields] OR "targetable"[All Fields] OR "targeted"[All Fields] OR "targeting"[All Fields] OR "targetings"[All Fields] OR "targets"[All Fields] OR "targetted"[All Fields] OR "targetting"[All Fields]) AND ("surgery"[MeSH Subheading] OR "surgery"[All Fields] OR "surgical procedures, operative"[MeSH Terms] OR ("surgical"[All Fields] AND "procedures"[All Fields] AND "operative"[All Fields]) OR "operative surgical procedures"[All Fields] OR "general surgery"[MeSH Terms] OR ("general"[All Fields] AND "surgery"[All Fields]) OR "general surgery"[All Fields] OR "surgery s"[All Fields] OR "surgerys"[All Fields] OR "surgeries"[All Fields])) OR (("select"[All Fields] OR "selectability"[All Fields] OR "selectable"[All Fields] OR "selected"[All Fields] OR "selecting"[All Fields] OR "selection s"[All Fields] OR "selection, genetic"[MeSH Terms] OR ("selection"[All Fields] AND "genetic"[All Fields]) OR "genetic selection"[All Fields] OR "selection"[All Fields] OR "selectional"[All Fields] OR "selections"[All Fields] OR "selective"[All Fields] OR "selectively"[All Fields] OR "selectives"[All Fields] OR "selectivities"[All Fields] OR "selectivity"[All Fields] OR "selects"[All Fields]) AND ("axilla"[MeSH Terms] OR "axilla"[All Fields] OR "axillary"[All Fields] OR "axillaries"[All Fields] OR "axillaris"[All Fields]) AND ("dissect"[All Fields] OR "dissected"[All Fields] OR "dissecting"[All Fields] OR "dissection"[MeSH Terms] OR "dissection"[All Fields] OR "dissections"[All Fields] OR "dissects"[All Fields])) OR ("marked"[All Fields] AND ("lymph node excision"[MeSH Terms] OR ("lymph"[All Fields] AND "node"[All Fields] AND "excision"[All Fields]) OR "lymph node excision"[All Fields] OR ("lymph"[All Fields] AND "node"[All Fields] AND "removal"[All Fields]) OR "lymph node removal"[All Fields])) OR (("clipped"[All Fields] OR "clipping"[All Fields] OR "clippings"[All Fields] OR "surgical instruments"[MeSH Terms] OR ("surgical"[All Fields] AND "instruments"[All Fields]) OR "surgical instruments"[All Fields] OR "clips"[All Fields]) AND "node"[All Fields] AND ("excisable"[All Fields] OR "excise"[All Fields] OR "excised"[All Fields] OR "excises"[All Fields] OR "excising"[All Fields] OR "excision"[All Fields] OR "excisions"[All Fields])) OR (("sentinel"[All Fields] OR "sentinels"[All Fields]) AND ("lymph node excision"[MeSH Terms] OR ("lymph"[All Fields] AND "node"[All Fields] AND "excision"[All Fields]) OR "lymph node excision"[All Fields] OR "lymphadenectomies"[All Fields] OR "lymphadenectomy"[All Fields])) OR (("target"[All Fields] OR "targetability"[All Fields] OR "targetable"[All Fields] OR "targeted"[All Fields] OR "targeting"[All Fields] OR "targetings"[All Fields] OR "targets"[All Fields] OR "targetted"[All Fields] OR "targetting"[All Fields]) AND ("lymph nodes"[MeSH Terms] OR ("lymph"[All Fields] AND "nodes"[All Fields]) OR "lymph nodes"[All Fields] OR ("lymph"[All Fields] AND "node"[All Fields]) OR "lymph node"[All Fields]) AND ("biopsie"[All Fields] OR "biopsy"[MeSH Terms] OR "biopsy"[All Fields] OR "biopsied"[All Fields] OR "biopsies"[All Fields] OR "biopsy s"[All Fields] OR "biopsying"[All Fields] OR "biopsys"[All Fields] OR "pathology"[MeSH Subheading] OR "pathology"[All Fields])) OR ("tad"[All Fields] AND ("methods"[MeSH Terms] OR "methods"[All Fields] OR "procedure"[All Fields] OR "methods"[MeSH Subheading] OR "procedures"[All Fields] OR "procedural"[All Fields] OR "procedurally"[All Fields] OR "procedure s"[All Fields])) OR ("dual-tracer"[All Fields] AND ("methods"[MeSH Terms] OR "methods"[All Fields] OR "technique"[All Fields] OR "methods"[MeSH Subheading] OR "techniques"[All Fields] OR "technique s"[All Fields]))) AND ((("complete"[All Fields] OR "completed"[All Fields] OR "completely"[All Fields] OR "completeness"[All Fields] OR "completer"[All Fields] OR "completers"[All Fields] OR "completes"[All Fields] OR "completing"[All Fields] OR "completion"[All Fields] OR "completions"[All Fields]) AND

("axilla"[MeSH Terms] OR "axilla"[All Fields] OR "axillary"[All Fields] OR "axillaries"[All Fields] OR "axillaris"[All Fields]) AND ("dissect"[All Fields] OR "dissected"[All Fields] OR "dissecting"[All Fields] OR "dissection"[MeSH Terms] OR "dissection"[All Fields] OR "dissections"[All Fields] OR "dissects"[All Fields])) OR (("radical"[All Fields] OR "radical s"[All Fields] OR "radicals"[All Fields]) AND ("axilla"[MeSH Terms] OR "axilla"[All Fields] OR "axillary"[All Fields] OR "axillaries"[All Fields] OR "axillaris"[All Fields]) AND ("surgery"[MeSH Subheading] OR "surgery"[All Fields] OR "surgical procedures, operative"[MeSH Terms] OR ("surgical"[All Fields] AND "procedures"[All Fields] AND "operative"[All Fields]) OR "operative surgical procedures"[All Fields] OR "general surgery"[MeSH Terms] OR ("general"[All Fields] AND "surgery"[All Fields]) OR "general surgery"[All Fields] OR "surgery s"[All Fields] OR "surgerys"[All Fields] OR "surgeries"[All Fields])) OR (("level"[All Fields] OR "levels"[All Fields]) AND "i-ii"[All Fields] AND ("dissect"[All Fields] OR "dissected"[All Fields] OR "dissecting"[All Fields] OR "dissection"[MeSH Terms] OR "dissection"[All Fields] OR "dissections"[All Fields] OR "dissects"[All Fields])) OR (("axilla"[MeSH Terms] OR "axilla"[All Fields] OR "axillary"[All Fields] OR "axillaries"[All Fields] OR "axillaris"[All Fields]) AND ("clearance"[All Fields] OR "clearances"[All Fields])) OR ("standalone"[All Fields] AND "SLNB"[All Fields]) OR ("biopsie"[All Fields] OR "biopsy"[MeSH Terms] OR "biopsy"[All Fields] OR "biopsied"[All Fields] OR "biopsies"[All Fields] OR "biopsy s"[All Fields] OR "biopsying"[All Fields] OR "biopsys"[All Fields] OR "pathology"[MeSH Subheading] OR "pathology"[All Fields]) OR (("conventional"[All Fields] OR "conventionals"[All Fields]) AND ("axilla"[MeSH Terms] OR "axilla"[All Fields] OR "axillary"[All Fields] OR "axillaries"[All Fields] OR "axillaris"[All Fields]) AND ("surgery"[MeSH Subheading] OR "surgery"[All Fields] OR "surgical procedures, operative"[MeSH Terms] OR ("surgical"[All Fields] AND "procedures"[All Fields] AND "operative"[All Fields]) OR "operative surgical procedures"[All Fields] OR "general surgery"[MeSH Terms] OR ("general"[All Fields] AND "surgery"[All Fields]) OR "general surgery"[All Fields] OR "surgery s"[All Fields] OR "surgerys"[All Fields] OR "surgeries"[All Fields]))) AND ((("lymph nodes"[MeSH Terms] OR ("lymph"[All Fields] AND "nodes"[All Fields]) OR "lymph nodes"[All Fields] OR ("lymph"[All Fields] AND "node"[All Fields]) OR "lymph node"[All Fields]) AND ("assess"[All Fields] OR "assessed"[All Fields] OR "assessement"[All Fields] OR "assesses"[All Fields] OR "assessing"[All Fields] OR "assessment"[All Fields] OR "assessment s"[All Fields] OR "assessments"[All Fields])) OR (("axilla"[MeSH Terms] OR "axilla"[All Fields] OR "axillary"[All Fields] OR "axillaries"[All Fields] OR "axillaris"[All Fields]) AND ("stage"[All Fields] OR "staged"[All Fields] OR "stages"[All Fields] OR "staging"[All Fields] OR "stagings"[All Fields])) OR (("nodal"[All Fields] OR "nodally"[All Fields] OR "nodals"[All Fields]) AND "status"[All Fields] AND ("accuracies"[All Fields] OR "accuracy"[All Fields])) OR (("pathologic"[All Fields] OR "pathologically"[All Fields] OR "pathologics"[All Fields] OR "pathology"[MeSH Terms] OR "pathology"[All Fields] OR "pathological"[All Fields]) AND ("stage"[All Fields] OR "staged"[All Fields] OR "stages"[All Fields] OR "staging"[All Fields] OR "stagings"[All Fields])) OR (("sensitive"[All Fields] OR "sensitively"[All Fields] OR "sensitives"[All Fields] OR "sensitivities"[All Fields] OR "sensitivity and specificity"[MeSH Terms] OR ("sensitivity"[All Fields] AND "specificity"[All Fields]) OR "sensitivity and specificity"[All Fields] OR "sensitivity"[All Fields]) AND ("j rehabil assist technol eng"[Journal] OR "rate"[All Fields])) OR (("identifed"[All Fields] OR "identification, psychological"[MeSH Terms] OR ("identification"[All Fields] AND "psychological"[All Fields]) OR "psychological identification"[All Fields] OR "identification"[All Fields] OR "identifications"[All Fields]) AND ("j rehabil assist technol eng"[Journal] OR "rate"[All Fields])) OR ("miss"[All Fields] AND ("j rehabil assist technol eng"[Journal] OR "rate"[All Fields])) OR (("diagnosis"[MeSH Terms] OR "diagnosis"[All Fields] OR "diagnostic"[All Fields] OR "diagnostical"[All Fields] OR "diagnostically"[All Fields] OR "diagnostics"[All Fields]) AND ("yield"[All Fields] OR "yielded"[All Fields] OR "yielding"[All Fields] OR "yields"[All Fields])))

2. Scopus

Scopus uses the TITLE-ABS-KEY field for searching and focuses on free-text terms. Here's the adaptation:

N = 140

TITLE-ABS-KEY (((Breast cancer) OR (mammary cancer) OR (breast malignancy) OR (breast adenocarcinoma) OR (carcinoma of the breast) AND (node-positive OR nodal involvement) OR (positive lymph node*) OR (axillary metastasis) OR (metastatic lymph node*)) AND ((node-negative) OR (nodal clearance) OR (negative lymph node*) OR (axillary response) OR (pathologic node-negative)) AND ((neoadjuvant therapy) OR (neoadjuvant chemotherapy) OR (preoperative therapy) OR (induction chemotherapy) OR (primary systemic therapy)) AND ((axillary targeted surgery) OR (selective axillary dissection) OR (marked lymph node removal) OR (clipped node excision) OR (sentinel lymphadenectomy) OR (targeted lymph node biopsy) OR (TAD procedure) OR (dual-tracer technique)) AND ((complete axillary dissection) OR (radical axillary surgery) OR (level I-II dissection) OR (axillary clearance) OR (standalone SLNB) OR (sentinel-only biopsy) OR (conventional axillary surgery)) AND ((lymph node

assessment) OR (axillary staging) OR (nodal status accuracy) OR (pathologic staging) OR (sensitivity rate) OR (identification rate) OR (miss rate) OR (diagnostic yield)))

3. CENTRAL (Cochrane Library)

CENTRAL accepts Boolean operators but does not support indexing like MeSH or Emtree. Focus on free-text terms

N = 50 trials.

((Breast cancer) OR (mammary cancer) OR (breast malignancy) OR (breast adenocarcinoma) OR (carcinoma of the breast) AND (node-positive OR nodal involvement) OR (positive lymph node*) OR (axillary metastasis) OR (metastatic lymph node*)) AND ((node-negative) OR (nodal clearance) OR (negative lymph node*) OR (axillary response) OR (pathologic node-negative)) AND ((neoadjuvant therapy) OR (neoadjuvant chemotherapy) OR (preoperative therapy) OR (induction chemotherapy) OR (primary systemic therapy)) AND ((axillary targeted surgery) OR (selective axillary dissection) OR (marked lymph node removal) OR (clipped node excision) OR (sentinel lymphadenectomy) OR (targeted lymph node biopsy) OR (TAD procedure) OR (dual-tracer technique)) AND ((complete axillary dissection) OR (radical axillary surgery) OR (level I-II dissection) OR (axillary clearance) OR (standalone SLNB) OR (sentinel-only biopsy) OR (conventional axillary surgery)) AND ((lymph node assessment) OR (axillary staging) OR (nodal status accuracy) OR (pathologic staging) OR (sensitivity rate) OR (identification rate) OR (miss rate) OR (diagnostic yield)) in Title Abstract Keyword - (Word variations have been searched)

4. Web of Science

Web of Science uses TS= for topic searches (title, abstract, and keywords):

N= 120

From advanced search: ((Breast cancer) OR (mammary cancer) OR (breast malignancy) OR (breast adenocarcinoma) OR (carcinoma of the breast) AND (node-positive OR nodal involvement) OR (positive lymph node*) OR (axillary metastasis) OR (metastatic lymph node*)) (All Fields) and ((node-negative) OR (nodal clearance) OR (negative lymph node*) OR (axillary response) OR (pathologic node-negative)) (All Fields) and ((neoadjuvant therapy) OR (neoadjuvant chemotherapy) OR (preoperative therapy) OR (induction chemotherapy) OR (primary systemic therapy)) (All Fields) and ((axillary targeted surgery) OR (selective axillary dissection) OR (marked lymph node removal) OR (clipped node excision) OR (sentinel lymphadenectomy) OR (targeted lymph node biopsy) OR (TAD procedure) OR (dual-tracer technique)) (All Fields) and ((complete axillary dissection) OR (radical axillary surgery) OR (level I-II dissection) OR (axillary clearance) OR (standalone SLNB) OR (sentinel-only biopsy) OR (conventional axillary surgery)) (All Fields) and ((axillary staging) OR (nodal status accuracy) OR (pathologic staging) OR (sensitivity rate) OR (identification rate) OR (miss rate) OR (diagnostic yield)) (All Fields)

Table S1. Clinicopathologic characteristics of included studies.

Study ID	TNM Stage	Tumor type		Initial Nodal Status	Neoadjuvant Therapy Type	Clinical Node-Negative Criteria	Complications (%)
		histological types	molecular types				
Acea-Figueira et al., 2023 (30)	T1-3N1M0	NR	Luminal A, Luminal B HER2-, Luminal B HER2 positive, HER2 positive, triple negative	cN1	Chemotherapy, endocrine, targeted therapy	physical examination and imaging	NR
Aguirre et al., 2022 (31)	T1-3 N0-2 M0	ICD: 90.0%, ILC: 2.5%, Other types: 7.5%	Luminal A: 23.8%<br>- Luminal B/HER2-negative: 33.7%<br>- Luminal B/HER2-positive: 18.8%<br>- HER2-positive (non-luminal): 7.5%<br>- Triple-negative (basal-like): 16.2%	N0-2	chemotherapy	clinical	NR
Alarcón et al., 2021 (32)	cT1-4 cN+ M0	IDC: 78.6% others 21.4%	Luminal A 14.6%, Luminal B / HER2− 44.7%, Luminal B / HER2+ 16.5%, HER2+ (non-luminal) 9.7%, Triple Negative 13.6%, Mixed Pattern (LB–TN) 1%	cN+	Chemotherapy, targeted therapy	physical examination and imaging	0
Aragon-Sánchez et al., 2022 (33)	T1-3N1M0	IDC: 96.9%, ILC: 3.1%	.- Luminal B: 46.9%<br>- Luminal/HER2-positive: 21.9%<br>- Triple-negative: 18.7%<br>- Luminal A: 9.4%<br>- HER2-enriched: 3.1%	CN1	chemotherapy and targeted therapy	physical examination and imaging	NR
Balasubramanian et al., 2020 (34)	T+N1M0	NR	HER2-Positive 38%, Triple-Negative 29%, HR-Positive/HER2-Negative 32%	cN1	NACT with HER2 traget therapy	imaging	NR
Barry et al., 2023 (35)	T1-4N+M0	IDC: 85%, ILC: 9%, Mixed: 5%, Mucinous: 1%	NACT-TAD: ER+/HER2- 23%, ER+/HER2+ 23%, ER-/HER2+ 24%, TNBC 30%<br>Primary TAD: ER+/HER2- 86%, ER+/HER2+ 4%, ER-/HER2+ 2%, TNBC 7%	cN1/2 (NACT-TAD), cN1 (Primary TAD)	Chemotherapy	imaging	NR
Beniey et al., 2021 (36)	T1-4N+M0	IDC: 88.6%, ILC: 2.9%, invasive mixed carcinoma and invasive mammary carcinoma: 8.6%	HR+/HER2−: 60.0%<br>- HR+/HER2+: 20.0%<br>- HR−/HER2+: 8.6%<br>- Triple-negative: 11.4%	cN+	chemotherapy and targeted therapy	imaging and physical examination	2.86% axillary wound cellulitis
Boniface et al., 2022 (37)	cT1-4 cN+ M0	Ductal: 89.3%, Lobular: 6.7%, Mixed ductal and lobular: 1.3%, Others: 2.7%	HR-positive / HER2-negative 42.3%, HR-positive / HER2-positive 20.1% , HR-negative / HER2-positive 12.8% , Triple-negative 24.8%	cN+	Chemotherapy, targeted therapy	physical examination and imaging	NR
Boyle, 2025 (38)	cT1–3N1M0	IDC: 97.7%, Occult Carcinoma: 2.3%	HR-positive / HER2-negative 18.2%<br>HR-positive / HER2-positive 31.8%<br>HR-negative / HER2-positive 25.0%<br>Triple Negative (HR−/HER2−) 25.0%	cN+	Chemotherapy; HER2+ patients received trastuzumab/pertuzumab; triple-negative patients received pembrolizumab.	physical examination	NR

Castillo et al., 2023 (39)	cT1-4 cN+ M0	NR	Luminal A 23.6%, Luminal B 51.0%, HER2-positive 18.0%, Triple Negative 7.2%	cN+	Chemotherapy, targeted therapy	physical examination and imaging	NR
Caudle et al., 2016 (28)	cT0-4 cN+ M0	Ductal lobular	HR+/HER2− 58%, HR+/HER2+ 16%, HR−/HER2+ 8%, Triple Negative 18%	cN+	Chemotherapy (98%), endocrine therapy (2%), targeted therapy	NR	NR
Chen et al., 2024 (40)	cT1–3N1M0	IDC: 98.6%, ILC/ others: 1.4%	Luminal A 4.1%<br>Luminal B 35.6%<br>Luminal-HER2 24.7%<br>HER2+ (non-luminal) 27.4%<br>Triple-negative 8.2%	cN1	Chemotherapy, targeted therapy	physical examination and imaging	0
Dashevsky et al., 2018 (41)	T2–T4, N1-2, M0	IDC, ILC	HER2+, luminal A/B, and triple negative subtypes.	cN1-2	NACT with HER2 target therapy	imaging	0
Diego et al., 2016 (42)	T2-3N1M0	IDC: 97%, ILC: 3%	Molecular phenotype:<br>- HER2-positive: 57%<br>- Triple-negative: 36%<br>- Luminal (HR+/HER2−): 7%	cN1	chemotherapy, targeted therapy	Physical examination	NR
Dilege et al., 2023 (43)	T1-3N+M0	NR	ER/PR-positive, HER2-negative: (60%)<br>- ER/PR-positive, HER2-positive: (20%)<br>- ER/PR-negative, HER2-positive: (13.3%)<br>- Triple-negative (6.7%)	cN1-2	chemotherapy and targeted therapy	physical examination and imaging	NR
Dostalek et al., 2021 (44)	T0-2N0M0	Invasive carcinoma of NST: 89%, ILC: 11%	Estrogen receptor-positive (ER+): (40%)<br>- Progesterone receptor-positive (PR+): (24%)<br>- HER2/neu-positive: (19%)	cN+	chemotherapy and targeted therapy	imaging and surgical findings	NR
Dux et al., 2023 (45)	cTis–T4N1M0	ILC, IDC, and Invasive mammary	ER+/HER2− 38%, HER2 Positive 40%, Triple Negative 22%	cN+	Chemotherapy, targeted therapy	NR	NR
Flores-Funes et al., 2021 (46)	T1-3N1M0	Infiltrating ductal carcinoma, Infiltrating lobular carcinoma, Infiltrating (NST)	Luminal A, Luminal B HER2 +, Luminal B HER2-, HER2 +,Triple negative	cN1	chemotherapy and targeted therapy	imaging	0
Gallagher et al., 2022 (47)	cT1-2 cN+ M0	NR	HR-positive / HER2-negative 50.6% , HR-positive / HER2-positive 15.3% , HR-negative / HER2-positive 8.2%, Triple-negative 25.9%	cN1-3	chemotherapy, endocrine, target therapy	Not explicitly noted	15.7% post-placement pain; allergic reaction (1.2%)
García-Novoa et al., 2020 (48)	T1-3N1M0	NR	Luminal A, Luminal B HER2-, Luminal B HER2 positive, HER2 positive, triple negative	cN1	chemotherapy	Imaging	NR
Gurleyik et al., 2021 (49)	T1–T3, N1, M0	IDC and ILC	Luminal A, Luminal B, triple negative, HER2	cN1	chemotherapy and targeted therapy	physical examination and imaging	NR
Hartmann et al., 2018 (50)	cT1-3N1-3M0	IDC: 83.3%, ILC: 10.0%	IHR+/HER2−, HR+/HER2+, Triple-negative, HR−/HER2+	cN1-3	chemotherapy and targeted therapy	imaging	NR

Kaya et al., 2024 (51)	cT1-3 cN+ M0	IDC: 81.9%, ILC: 9.6%, Others: 8.4%	Luminal A (65.1%) Luminal B (12.0%) HR+/HER2+ (8.4%) HR−/HER2+ (8.4%) Triple Negative (TN) (6.0%)	cN+	Chemotherapy	physical examination and imaging	(3.6%) Minor hematoma during clip placement
Kim et al., 2019 (52)	T1-4N1-3M0	NR	Hormone receptor–positive (HR+) / HER2-negative: (61%) - HR-negative / HER2-positive: (29%) - Triple-negative (ER−/PR−/HER2−): (11%)	cN+	chemotherapy and targeted therapy	imaging	NR
Kuemmel et al., 2022 (53)	cT1-4 cN+ M0	NR	HR+/HER2− 43.3%, HR+/HER2+ 19.7%, HR−/HER2+ 14.0%, Triple-Negative 23%	cN+	Chemotherapy, endocrine therapy, targeted therapy	physical examination and imaging	NR
Kuemmel et al., 2023 (54)	cT1-4 cN+ M0	NR	HR+/HER2+ (ERBB2+) 19.6%, HR+/HER2− 44.2%, HR−/HER2+ (ERBB2+) 14.3% , Triple-Negative (HR−/HER2−) 22.0%	cN+	chemotherapy, endocrine, target therapy	physical examination and imaging	NR
Laws et al., 2020 (55)	T1-4N1-2M0	NR	ER-positive / HER2-negative: (28.1%) - ER-negative / HER2-negative: (26.3%) - ER-positive / HER2-positive: (29.8%) - ER-negative / HER2-positive: (15.8%)	cN1-2	chemotherapy, endocrine therapy and targeted therapy	physical examination and imaging	Axillary Seroma 14.3%, Axillary Surgical Site Infection 10.7%, Bleeding Requiring Reoperation 0%
Laws et al., 2025 (56)	cT1-4 cN+ M0	Ductal carcinoma, Lobular/mixed, others	HR-positive / HER2-negative, HR-positive / HER2-positive, HR-negative / HER2-positive, Triple Negative	cN1	chemotherapy	physical examination and imaging	NR
Loveland-Jones et al., 2024 (57)	cT0-4 cN+ M0	Ductal: 88.2%, Lobular: 2.7%, Mixed: 6.4, Others: 2.7%	HR-positive / HER2-negative 10.7%, HR-negative / HER2-negative 50.0%, HR-positive / HER2-positive 50%, HR-negative / HER2-positive 55.6%	cN+	Chemotherapy	physical examination and imaging	NR
Manuel et al., 2022 (58)	T1-3N1M0	IDC: 93.3%, Others: 6.6%	HR+/HER2− (Luminal A/B): (50%) - HR+/HER2+ (Luminal HER2): (16.6%) - HR−/HER2+ (HER2-enriched): (6.6%) - Triple-negative (TNBC): (26.6%)	CN1	chemotherapy and targeted therapy and endocrine therapy	physical examination and imaging	NR
Martínez et al., 2021 (59)	T1c-3N1-2M0	IDC: 93.4%, ILC: 3.3%, Others: 3.3%	Luminal A-like: (10%) - Luminal B-like HER2-negative: (46.7%) - Luminal B-like HER2-positive: (20%) - HER2-positive (non-luminal): (10%) - Triple-negative: 4 patients (13.3%)	cN+	chemotherapy and targeted therapy	physical examination and imaging	0
Martínez et al., 2022 (60)	cT1-3 cN+ M0	-	Luminal B (LB) 50.7%, HER2+/EP+ 33.3%, Triple Negative 12.3%, Luminal A 2.5%, LB/HER2+ 1.2%	cN+	Chemotherapy	physical examination and imaging	NR
Munck et al., 2023 (61)	T1-3N1M0	IDC: 90.3%, ILC: 2.0%, NOS: 7.7%	ER−/Her2− (19.7%) ER−/Her2+ (15.7%)	cN1	chemotherapy, targeted therapy	imaging	NR

			ER+/Her2− (39.4%) ER+/Her2+ (25.2%)				
Munck et al., 2023 (62)	T1-2N1M0	IDC: 88.7%, ILC:1 patient, DCIS: 2 patients, Others: 13 patients	ER+/HER2+: 38 patients (26.8%) - ER+/HER2−: 54 patients (38.0%) - ER−/HER2+: 27 patients (19.0%) - Triple-negative (ER−/PR−/HER2−): 23 patients (16.2%)	cN1	chemotherapy and targeted therapy	physical examination and imaging	NR
Muslumanoglu et al., 2025 (63)	cT1-4 cN+ M0	IDC, ILC, Mixed, and Others	HR-positive / HER2-negative 52.7%, HR-positive / HER2-positive 19.2%, HR-negative / HER2-positive 5.8%, Triple Negative 8.8%, Luminal A 13.6%	cN+	Chemotherapy, targeted therapy	physical examination and imaging	NR
Patel et al., 2019 (64)	T0-4 N0-3 M0	NR	HR+,HER2- (39.4%) HR-,HER2+ (15.2%) HR+,HER2+ (16.7%) HR-,HER2-: (28.8%)	cN+	Chemotherapy (Group2)	Not applicable	NR
Pfob et al., 2024 (65)	cT0-4 cN+ M0	NR	HR-positive / HER2-negative (40.2%) - HR-positive / HER2-positive (19.8%) - HR-negative / HER2-positive (10.3%) - Triple-Negative (16.7%) - Changing Receptor Status (13.1%)	cN+	Chemotherapy, targeted therapy	physical examination and imaging	NR
Pinto et al., 2022 (66)	T1-3N1M0	IDC: 97.9%, ILC: 2%	(ER/PR+), HER2-negative 51.6% - HER2-positive ±ER/PR 32.3% - Triple-negative 16.1%	cN1	chemotherapy and targeted therapy	imaging and surgical findings	NR
Pinto et al., 2022 (67)	T1–T3N1M0	NST: 94.6%,ILC: 2.7%, Others: 2.7%	HR+/HER2- (40.5%) HR+/HER2+ (32.4%) HR-/HER2+ (8.1%) Triple negative (18.9%)	cN1	chemotherapy, targeted therapy, and endocrine therapy	physical examination and imaging	2.7% acute allergic reaction to PBD dye
Plecha et al., 2015 (68)	T1-4, cN+,M0-1	IDC: 84.1%, ILC: 11.2%, Invasive Mammary Carcinoma: 3.7%, Others: 0.9%	NR	cN+	NACT	imaging	NR
Porpiglia et al., 2023 (69)	cT1-4 cN0-2 M0	IDC: 97.9%, ILC: 2%	ER+/HER2- (38.8%), HER2+ (38.8%), Triple-negative (22.4%)	cN+	chemotherapy and targeted therapy	clinical	NR
Reitsamer et al., 2021 (70)	cT1-4 cN+ M0	NR	Triple-Negative (30.0%) - HER2-Positive / ER−/PR− (30.0%) - HER2-Positive / ER+/PR+ (15.0%) - Luminal B-like (22.5%) - Luminal A-like (2.5%)	pN1	chemotherapy, targeted therapy	physical examination and imaging	0
Rella et al., 2023 (71)	T2-4N1-2M0	IDC: 58 patients, ILC: 9 patients, Others: 5 patients	Hormone receptor–positive / HER2–positive: 35 patients - Hormone receptor–positive / HER2–negative: 12 patients - Hormone receptor–negative / HER2–positive: 8 patients	cN1-2	chemotherapy and endocrine therapy	physical examination and imaging	NR

			- Triple-negative (HR– / HER2–): 17 patients				
Sierra et al, 2021 (72)	T1–T3, N1, M0	NR	Luminal A like, Luminal B like, HER2+, triple negative, Luminal B + HER2+	pN1+	chemotherapy, endocrine therapy, targeted therapy	imaging	NR
Simons et al., 2019 (73)	T1-4N+M0	IDC : 84.2%, ILC: 7.2%, Ductolobular carcinoma: 5.0%, Others: 3.6%	Hormone receptor-positive / HER2-negative: 48.9%<br>Hormone receptor-positive / HER2-positive: 17.3%<br>Hormone receptor-negative / HER2-positive: 15.8%<br>Triple-negative breast cancer: 18.0%	cN+	chemotherapy, targeted therapy and endocrine therapy	imaging and surgical findings	NR
Simons et al., 2021 (74)	T1-4N+M0	IDC: 94%, ILC: 6%	HR+/HER2−: (52%)<br>- HR+/HER2+: (20%)<br>- HR−/HER2+: (10%)<br>- Triple-negative (HR−/HER2−): (18%)	cN+	chemotherapy and targeted therapy	physical examination and imaging	0
Siso et al., 2018 (75)	cT1-4 cN+ M0	IDC: 91.3%, ILC: 8.7%	Luminal A 8.7%, Luminal B / HER2-negative 58.7%, Luminal B / HER2-positive 13%, HER2-enriched (non-luminal) 10.8%, Triple Negative 8.7%	cN+	Endocrine 6 (13.0)<br>Chemotherapy 29 (63.0)<br>Chemotherapy + anti-HER2 11 (23.9)	physical examination and imaging	NR
Siso et al., 2023 (76)	cT0-4 cN+ M0	Ductal<br>Lobular	Luminal A 22%, Luminal B HER2-negative 40% , Luminal B HER2-Positive 15%, HER2 Positive (Non-luminal) 14%, Triple Negative 9%	cN+	Chemotherapy, endocrine therapy, targeted therapy	physical examination and imaging	NR
Spautz et al., 2020 (77)	T1-4N1-2M0	IDC: 85.4%, ILC: 8.1%, Others: 6.5%	HR+/HER2- (47.9%)<br>HR+/HER2+ (22.8%)<br>HR-/HER2+ (8.9%)<br>HR-/HER2- (20.4%)	cN1-N2	chemotherapy and targeted therapy	imaging and physical examination	NR
Sun et al., 2021 (78)	T1-4N1-3M0	IDC: 100%	ER+/HER2−: 40%<br>- ER+/HER2+: 27%<br>- HER2+ only: 9%<br>- Triple-negative: 24%	cN1-3	chemotherapy and targeted therapy	physical examination and imaging	0
Sutton et al., 2020 (79)	cT1-4 cN+ M0	IDC: 96.6%, ILC: 3.4%	HR-positive 41.4%, HER2-positive 41.4% , Triple Negative 17.2%	cN1	Neoadjuvant chemotherapy	physical examination and imaging	NR
Taj et al., 2023 (80)	T1-3N1M0	NR	ER + or PR+, HER2- (52.5%)<br>ER - , PR - , HER2+ (18.75%)<br>Triple negative (15%)<br>Triple positive (13.75%)	cN1	endocrine therapy , chemotherapy , targeted therapy	imaging and physical examination	NR
Weinfurtner et al., 2022 (81)	T2-4N1-2M0	invasive breast carcinoma	ER and/or PR-positive: 39 patients<br>- HER2-positive: 37 patients<br>- Triple-negative (TNBC): 29 patients	cN+	chemotherapy , targeted therapy and endocrine therapy	physical examination and imaging	NR
Weiss et al., 2022 (82)	cT1-4 cN0-1 M0	IDC): 83%, ILC: 3%, Mixed: 8%, Others: 6%	HR+/HER2- 28%, HR+/HER2+ 32%, HR-/HER2+ 15%, TNBC 25%	cN0-1	chemotherapy	clinical + imaging	NR
Winder et al., 2022 (83)	T1-3N1-2M0	NR	ER-positive / HER2-negative: (28.9%)<br>- HER2-positive (ER-positive or ER-negative) : (36.8%)<br>- Triple-negative breast cancer (TNBC)	cN1-2	chemotherapy and targeted therapy	physical examination and imaging	3.5% lymphedema

			(34.2%)<br><br>Histologic Grade<br>- Grade 2 tumors: (42.1%)<br>- Grade 3 tumors: (57.9%)				
Wu et al., 2018 (84)	cT1-3 cN+ M0	IDC	HER2-positive , ER-positive , Triple-negative	cN+	Chemotherapy, targeted therapy	physical examination and imaging	NR
Wu et al., 2023 (85)	cT1-4 cN+ M0	NR	ER+/HER2− 27.0%, ER+/HER2+ 25.2%, ER−/HER2+ 27.3%, Triple-Negative 20.5%	cN1-3	Chemotherapy	physical examination and imaging	NR
Yang et al., 2023 (86)	cT1-3 cN+ M0	Ductal: 92.11%, lobular: 2.63%, Others: 5.26%	Luminal A 15.8%, Luminal B / HER2− 31.6% , Luminal B / HER2+ 18.4%, HER2+ (non-luminal) 23.7%, Triple Negative10.5%	cN+	Chemotherapy, targeted therapy	physical examination and imaging	NR
Yousri et al., 2025 (87)	cT1–3N1M0	IDC: 90%, ILC: 10%	Luminal A 13.3%<br>Luminal B 46.7%<br>HER2-enriched 20.0%<br>Triple Negative 20.0%	cN+	All patients received 8 cycles: 4 cycles of Adriamycin-based regimen followed by 4 cycles of Taxane-based regimen	physical examination and imaging	0

Abbreviations: CN, clinical nodal stage; CT, computed tomography; DCIS, ductal carcinoma in situ; ER, estrogen receptor; HER2, human epidermal growth factor receptor 2; HR, hormone receptor; ICD, invasive carcinoma of ductal origin; IDC, invasive ductal carcinoma; ILC, invasive lobular carcinoma; IQR, interquartile range; LN, lymph node; NACT, neoadjuvant chemotherapy; NOS, not otherwise specified; NST, no special type; PBD, patent blue dye; PR, progesterone receptor; SD, standard deviation; SLN, sentinel lymph node; TAD, targeted axillary dissection; TNBC, triple-negative breast cancer.

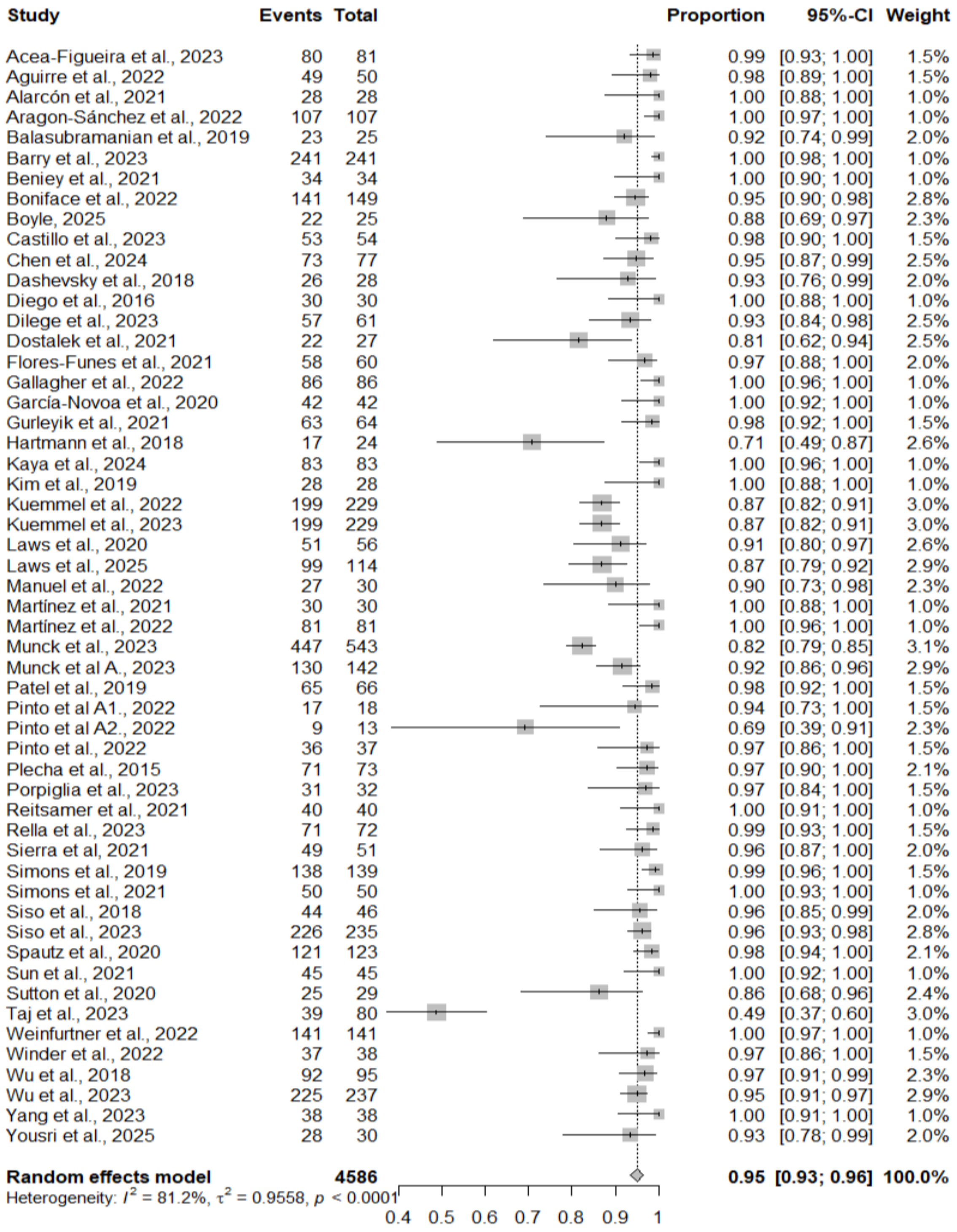

Figure S1: Forest plot of the pooled identification rate across the included studies.

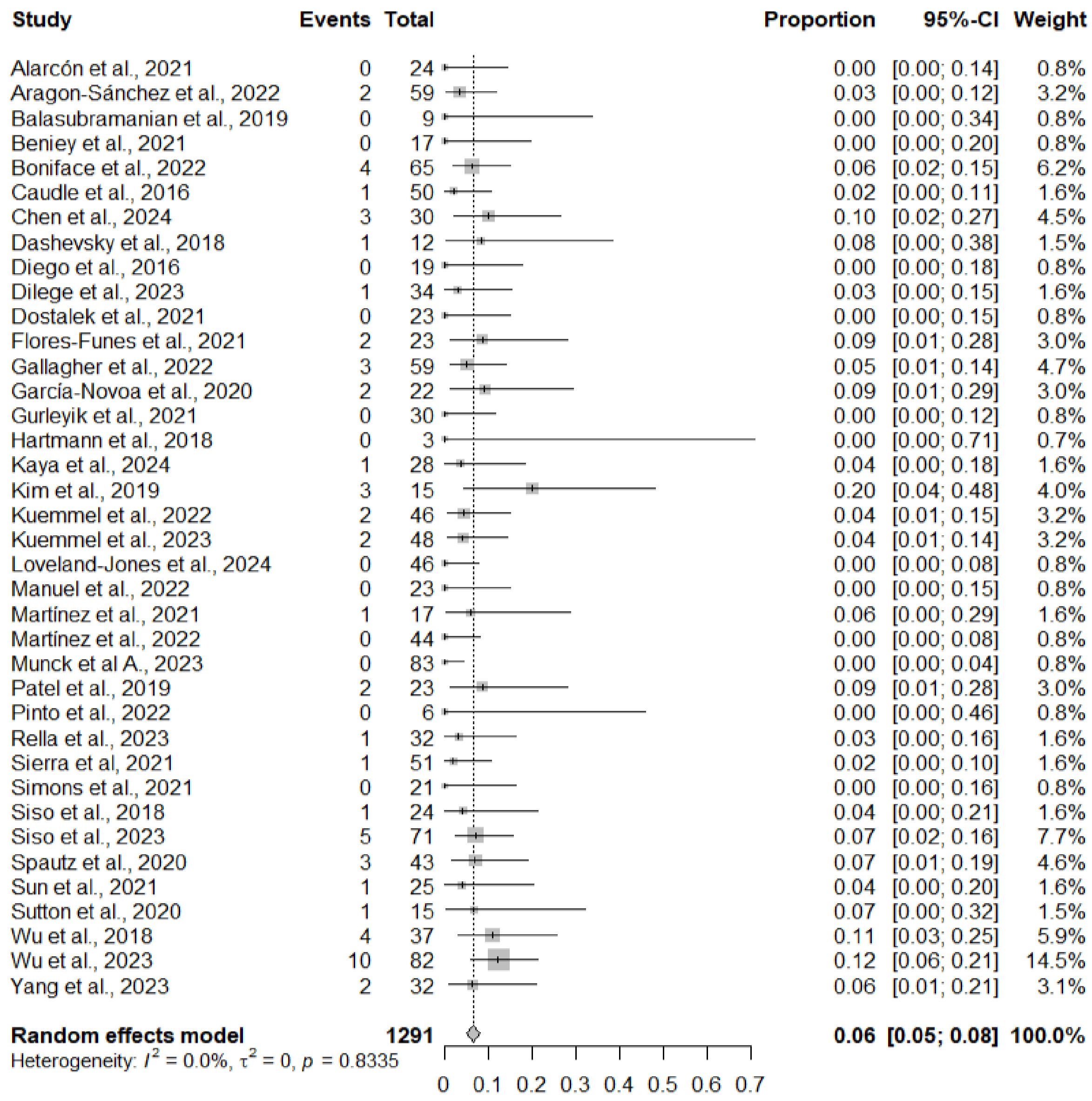

Figure S2. Forest plot of the pooled false negative rate (FNR) across the included studies.

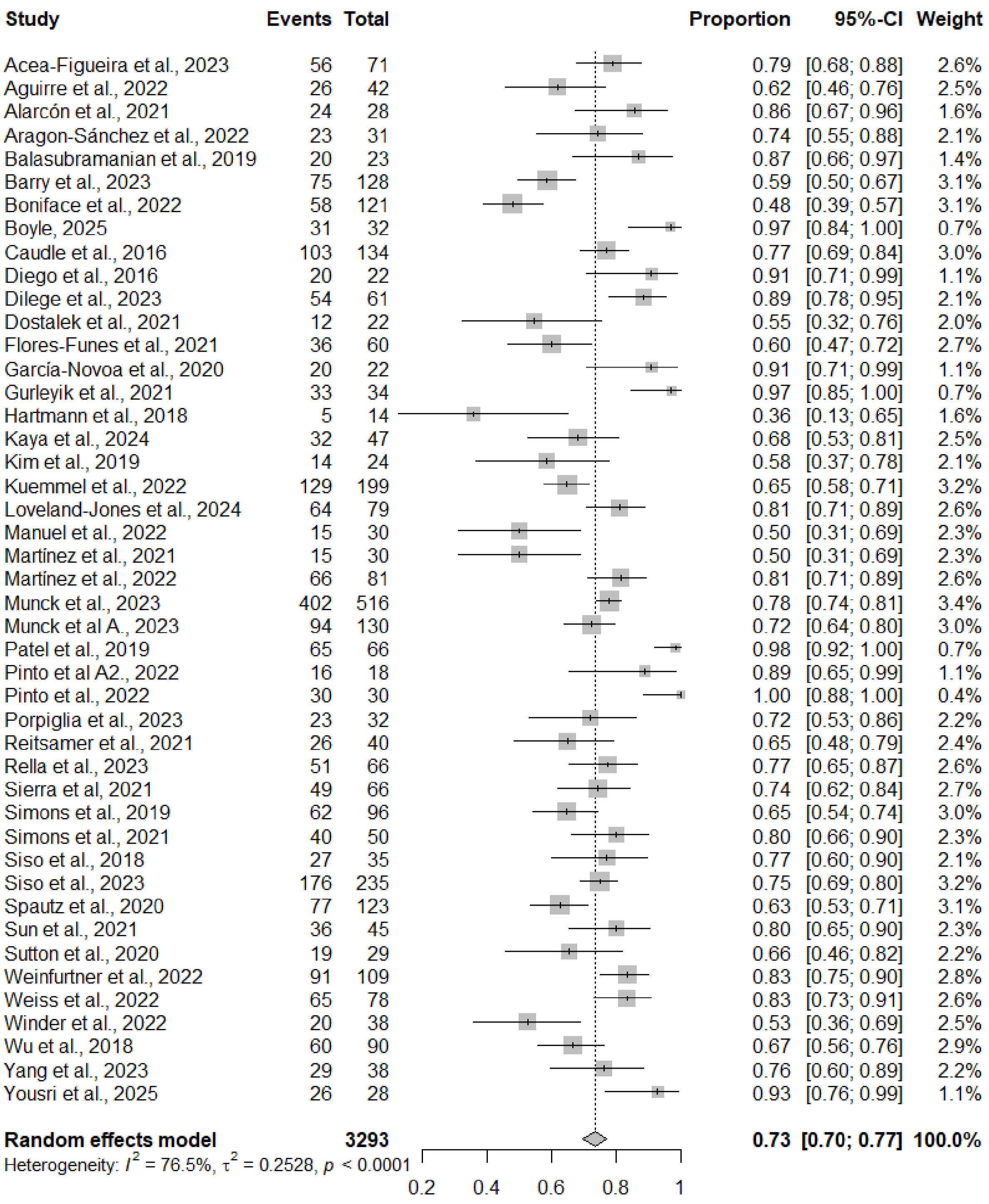

Figure S3. Forest plot of the pooled concordance rate across the included studies.

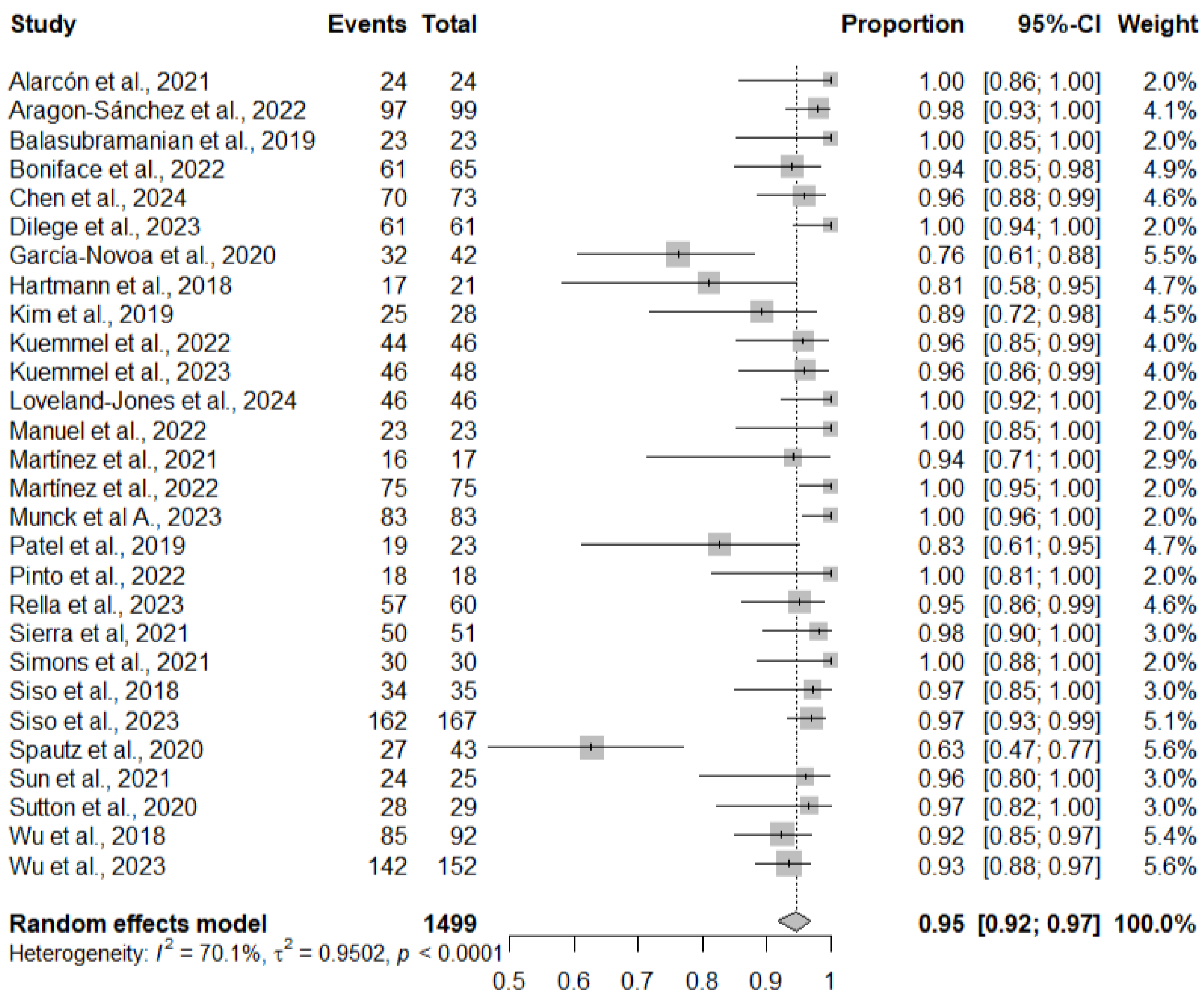

Figure S4. Forest plot of the pooled nodal staging accuracy rate across the included studies.

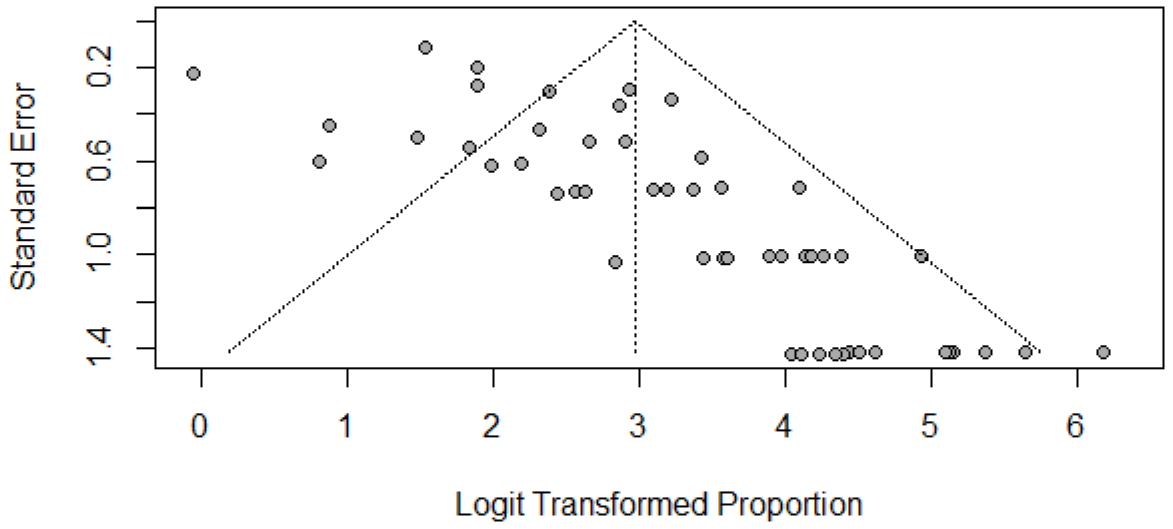

Figure S5. Funnel plot assessing publication bias for the identification rate.

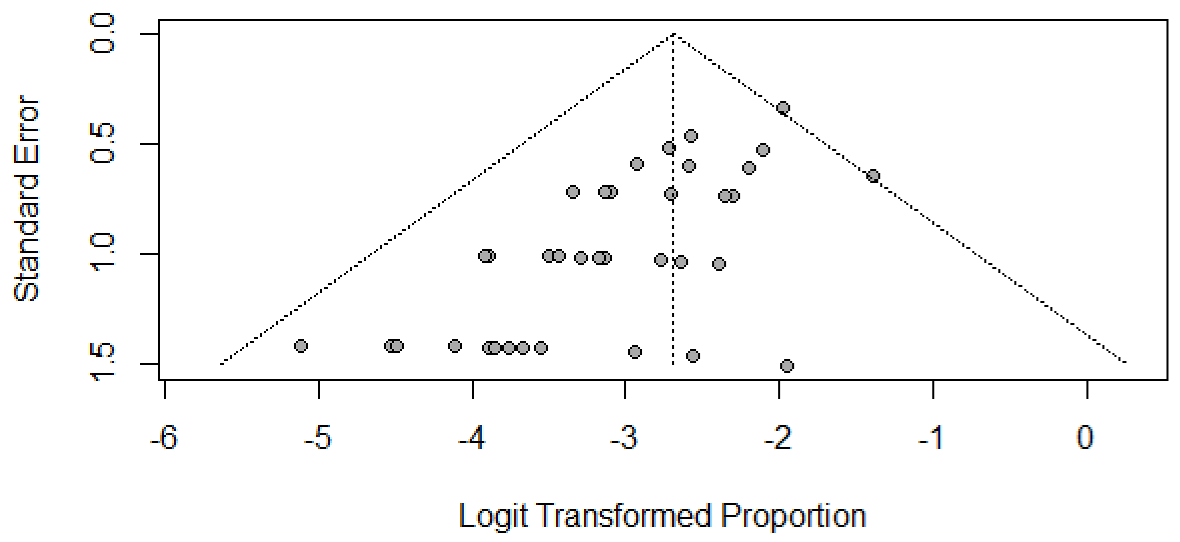

Figure S6. Funnel plot assessing publication bias for the False Negative Rate (FNR).

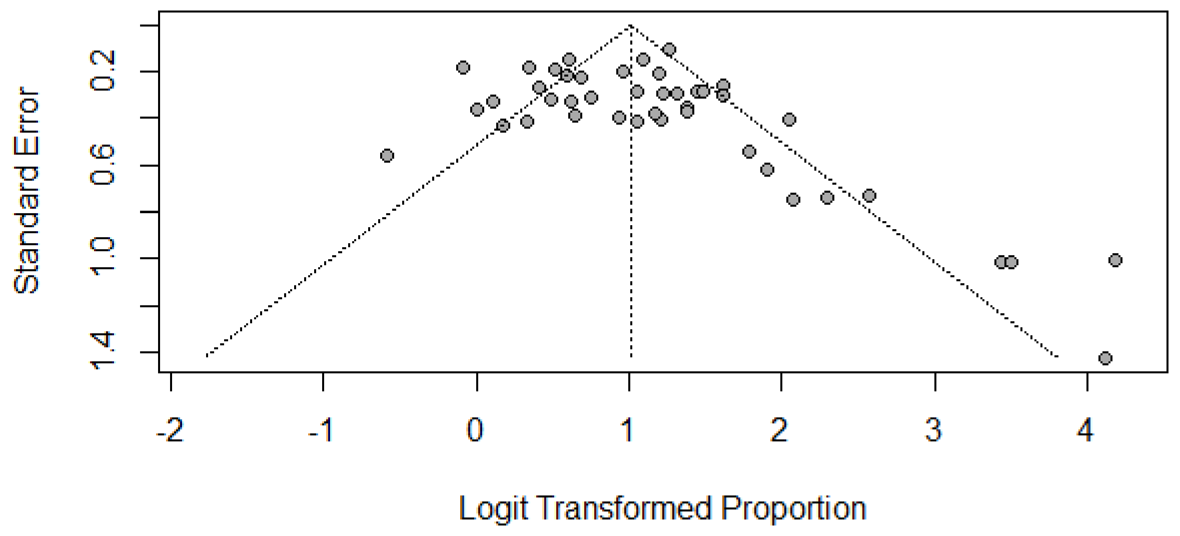

Figure S7. Funnel plot assessing publication bias for concordance rate.

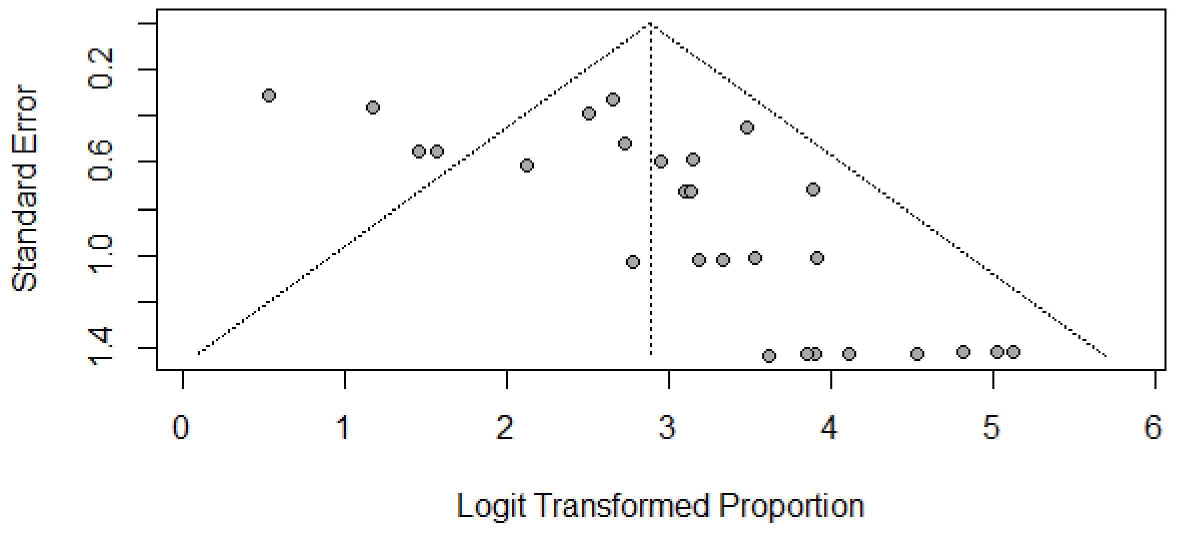

Figure S8. Funnel plot assessing publication bias for staging accuracy rate.

Study	Proportion	95%-CI	Tau2	Tau	I2
Omitting Acea-Figueira et al., 2023	0.95	[0.93; 0.96]	0.9414	0.9703	81.2%
Omitting Aguirre et al., 2022	0.95	[0.93; 0.96]	0.9500	0.9747	81.4%
Omitting Alarcón et al., 2021	0.95	[0.93; 0.96]	0.9538	0.9766	81.5%
Omitting Aragon-Sánchez et al., 2022	0.95	[0.93; 0.96]	0.9389	0.9690	81.2%
Omitting Balasubramanian et al., 2019	0.95	[0.93; 0.97]	0.9669	0.9833	81.6%
Omitting Barry et al., 2023	0.95	[0.93; 0.96]	0.9262	0.9624	81.0%
Omitting Beniey et al., 2021	0.95	[0.93; 0.96]	0.9521	0.9758	81.4%
Omitting Boniface et al., 2022	0.95	[0.93; 0.97]	0.9704	0.9851	81.2%
Omitting Boyle, 2025	0.95	[0.93; 0.97]	0.9718	0.9858	81.6%
Omitting Castillo et al., 2023	0.95	[0.93; 0.96]	0.9488	0.9741	81.4%
Omitting Chen et al., 2024	0.95	[0.93; 0.97]	0.9645	0.9821	81.4%
Omitting Dashevsky et al., 2018	0.95	[0.93; 0.97]	0.9660	0.9829	81.6%
Omitting Diego et al., 2016	0.95	[0.93; 0.96]	0.9532	0.9763	81.5%
Omitting Dilege et al., 2023	0.95	[0.93; 0.97]	0.9704	0.9851	81.5%
Omitting Dostalek et al., 2021	0.95	[0.93; 0.97]	0.9731	0.9864	81.5%
Omitting Flores-Funes et al., 2021	0.95	[0.93; 0.96]	0.9539	0.9767	81.4%
Omitting Gallagher et al., 2022	0.95	[0.93; 0.96]	0.9419	0.9705	81.3%
Omitting García-Novoa et al., 2020	0.95	[0.93; 0.96]	0.9501	0.9747	81.4%
Omitting Gurleyik et al., 2021	0.95	[0.93; 0.96]	0.9459	0.9726	81.3%
Omitting Hartmann et al., 2018	0.95	[0.94; 0.97]	0.9543	0.9769	81.2%
Omitting Kaya et al., 2024	0.95	[0.93; 0.96]	0.9423	0.9707	81.3%
Omitting Kim et al., 2019	0.95	[0.93; 0.96]	0.9538	0.9766	81.5%
Omitting Kuemmel et al., 2022	0.95	[0.94; 0.97]	1.0776	1.0381	81.6%
Omitting Kuemmel et al., 2023	0.95	[0.94; 0.97]	1.0776	1.0381	81.6%
Omitting Laws et al., 2020	0.95	[0.93; 0.97]	0.9787	0.9893	81.6%
Omitting Laws et al., 2025	0.95	[0.93; 0.97]	1.0181	1.0090	81.6%
Omitting Manuel et al., 2022	0.95	[0.93; 0.97]	0.9717	0.9858	81.6%
Omitting Martínez et al., 2021	0.95	[0.93; 0.96]	0.9532	0.9763	81.5%
Omitting Martínez et al., 2022	0.95	[0.93; 0.96]	0.9427	0.9709	81.3%
Omitting Munck et al., 2023	0.95	[0.94; 0.97]	1.1305	1.0633	79.7%
Omitting Munck et al A., 2023	0.95	[0.93; 0.97]	1.0036	1.0018	81.5%
Omitting Patel et al., 2019	0.95	[0.93; 0.96]	0.9453	0.9723	81.3%
Omitting Pinto et al A1., 2022	0.95	[0.93; 0.97]	0.9616	0.9806	81.6%
Omitting Pinto et al A2., 2022	0.95	[0.94; 0.97]	0.9549	0.9772	81.3%
Omitting Pinto et al., 2022	0.95	[0.93; 0.96]	0.9544	0.9769	81.4%
Omitting Plecha et al., 2015	0.95	[0.93; 0.96]	0.9491	0.9742	81.3%
Omitting Porpiglia et al., 2023	0.95	[0.93; 0.96]	0.9563	0.9779	81.5%
Omitting Reitsamer et al., 2021	0.95	[0.93; 0.96]	0.9506	0.9750	81.4%
Omitting Rella et al., 2023	0.95	[0.93; 0.96]	0.9437	0.9714	81.3%
Omitting Sierra et al, 2021	0.95	[0.93; 0.96]	0.9574	0.9785	81.4%
Omitting Simons et al., 2019	0.95	[0.93; 0.96]	0.9294	0.9640	81.0%
Omitting Simons et al., 2021	0.95	[0.93; 0.96]	0.9483	0.9738	81.4%
Omitting Siso et al., 2018	0.95	[0.93; 0.96]	0.9594	0.9795	81.5%
Omitting Siso et al., 2023	0.95	[0.93; 0.96]	0.9438	0.9715	80.7%
Omitting Spautz et al., 2020	0.95	[0.93; 0.96]	0.9329	0.9659	81.0%
Omitting Sun et al., 2021	0.95	[0.93; 0.96]	0.9494	0.9744	81.4%
Omitting Sutton et al., 2020	0.95	[0.93; 0.97]	0.9749	0.9874	81.6%
Omitting Taj et al., 2023	0.95	[0.94; 0.96]	0.6270	0.7918	72.6%
Omitting Weinfurtner et al., 2022	0.95	[0.93; 0.96]	0.9349	0.9669	81.2%
Omitting Winder et al., 2022	0.95	[0.93; 0.96]	0.9541	0.9768	81.4%
Omitting Wu et al., 2018	0.95	[0.93; 0.96]	0.9489	0.9741	81.2%
Omitting Wu et al., 2023	0.95	[0.93; 0.97]	0.9692	0.9845	80.9%
Omitting Yang et al., 2023	0.95	[0.93; 0.96]	0.9511	0.9752	81.4%
Omitting Yousri et al., 2025	0.95	[0.93; 0.97]	0.9654	0.9825	81.6%
**Random effects model**	**0.95**	**[0.93; 0.96]**	**0.9558**	**0.9776**	**81.2%**

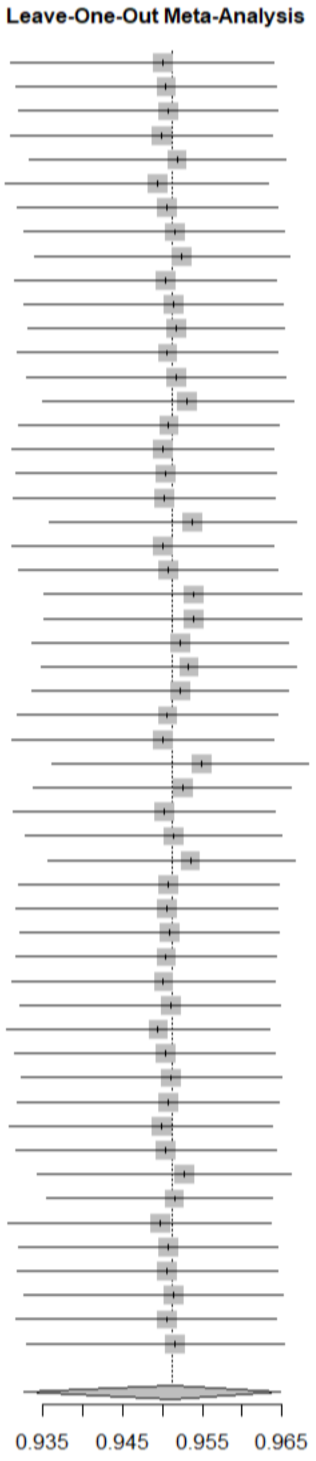

Figure S9. Leave-one-out sensitivity analysis for the identification rate.

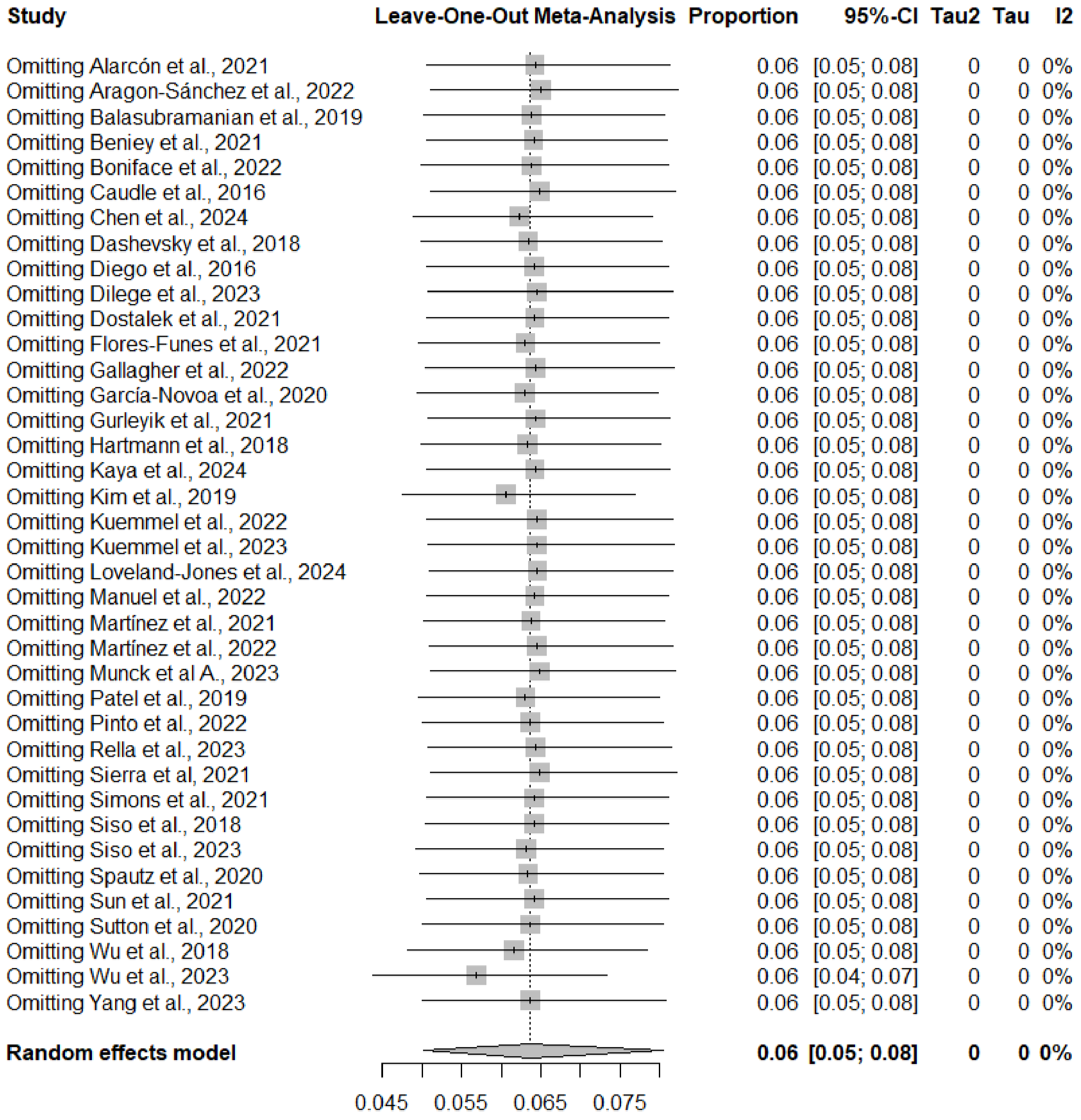

Figure S10. Leave-one-out sensitivity analysis for the false negative rate (FNR).

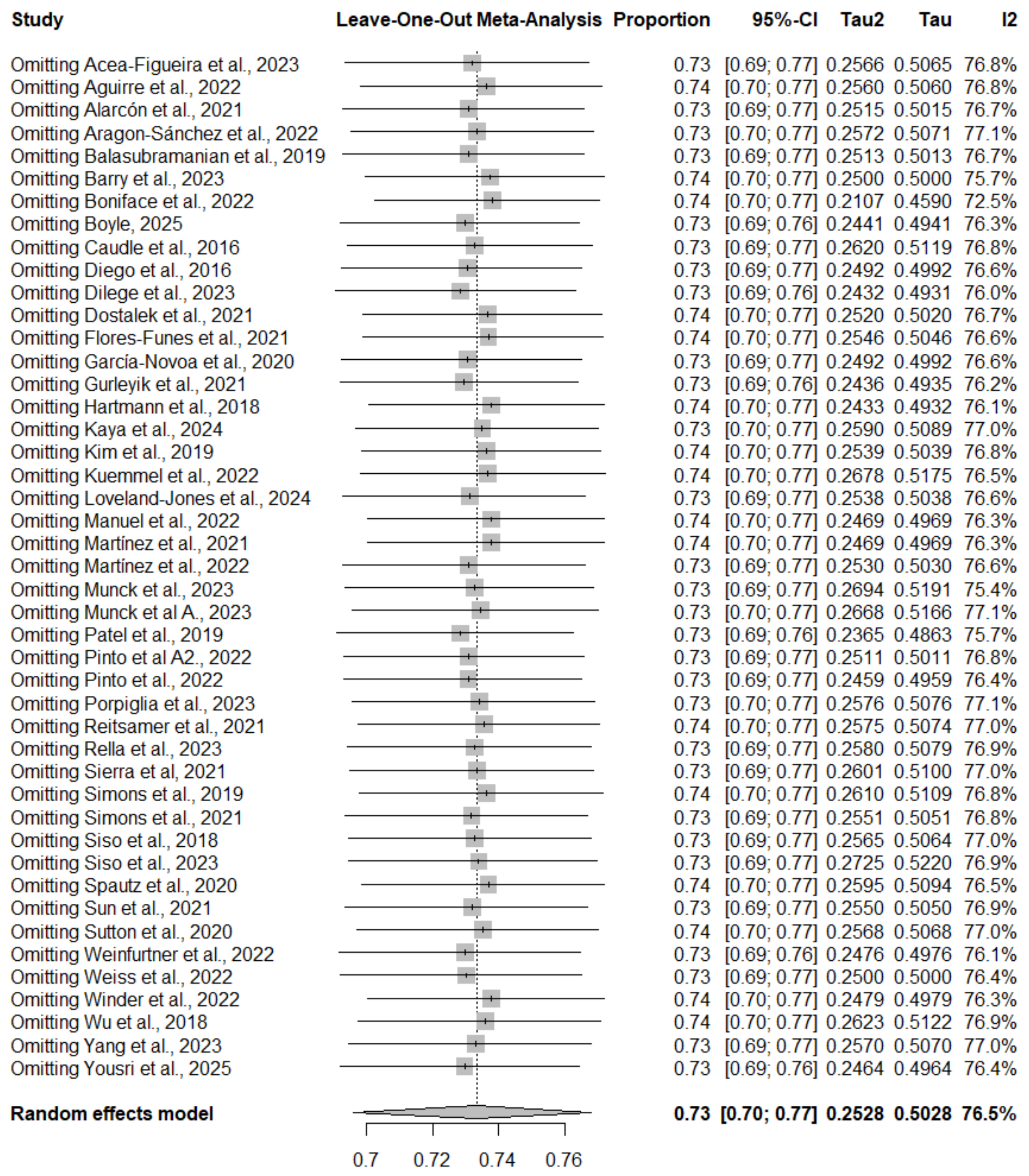

Figure S11. Leave-one-out sensitivity analysis for concordance rate.

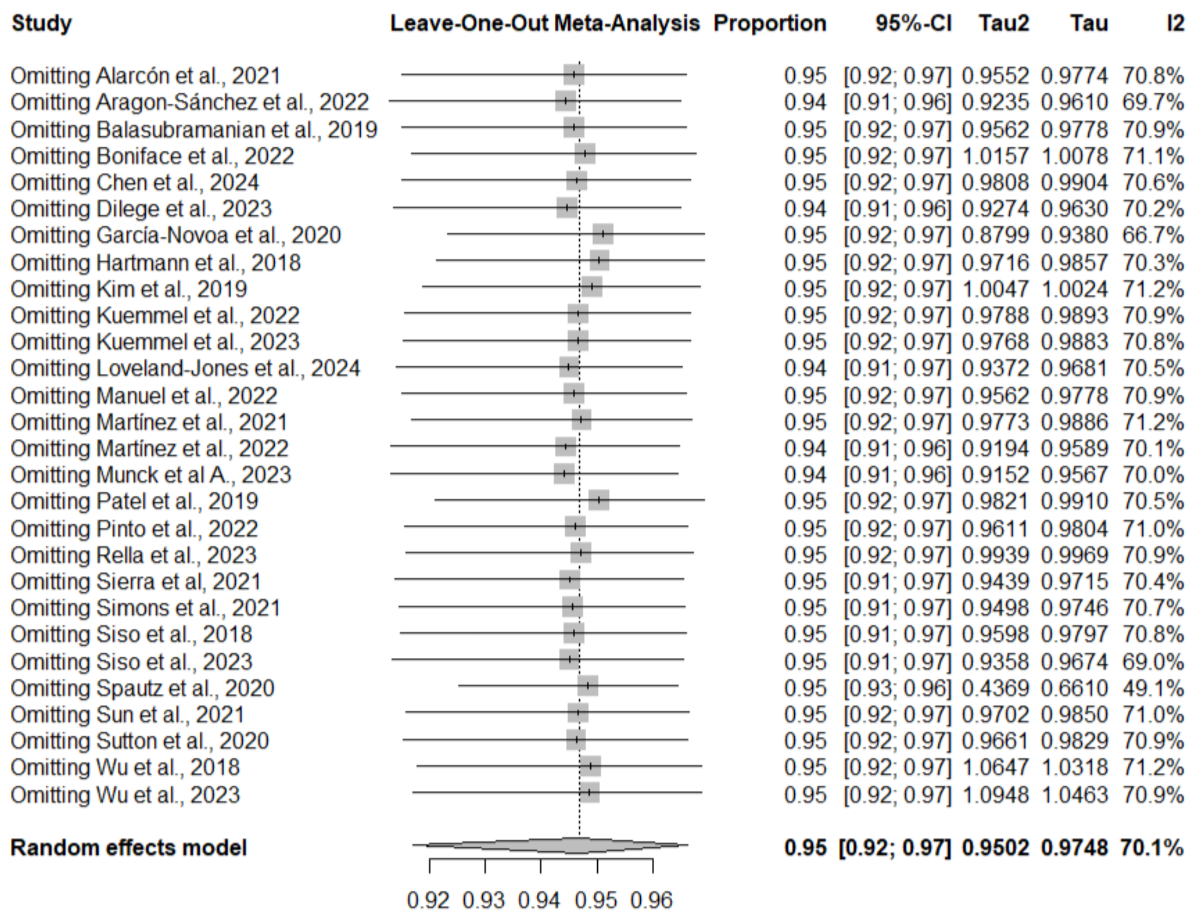

Figure S12. Leave-one-out sensitivity analysis for nodal staging accuracy rate.

Section and Topic	Item #	Checklist item	Location where item is reported
**TITLE**			
Title	1	Identify the report as a systematic review.	Page 1
**ABSTRACT**			
Abstract	2	See the PRISMA 2020 for Abstracts checklist.	Page 2
**INTRODUCTION**			
Rationale	3	Describe the rationale for the review in the context of existing knowledge.	Page 2, 3
Objectives	4	Provide an explicit statement of the objective(s) or question(s) the review addresses.	Page 4
**METHODS**			
Eligibility criteria	5	Specify the inclusion and exclusion criteria for the review and how studies were grouped for the syntheses.	Page 17, 18
Information sources	6	Specify all databases, registers, websites, organisations, reference lists and other sources searched or consulted to identify studies. Specify the date when each source was last searched or consulted.	Page 17
Search strategy	7	Present the full search strategies for all databases, registers and websites, including any filters and limits used.	Supplementary file
Selection process	8	Specify the methods used to decide whether a study met the inclusion criteria of the review, including how many reviewers screened each record and each report retrieved, whether they worked independently, and if applicable, details of automation tools used in the process.	Page 19
Data collection process	9	Specify the methods used to collect data from reports, including how many reviewers collected data from each report, whether they worked independently, any processes for obtaining or confirming data from study investigators, and if applicable, details of automation tools used in the process.	Page 17, 18, 19
Data items	10a	List and define all outcomes for which data were sought. Specify whether all results that were compatible with each outcome domain in each study were sought (e.g. for all measures, time points, analyses), and if not, the methods used to decide which results to collect.	Page 18
	10b	List and define all other variables for which data were sought (e.g. participant and intervention characteristics, funding sources). Describe any assumptions made about any missing or unclear information.	Page 16, 18, 19
Study risk of bias assessment	11	Specify the methods used to assess risk of bias in the included studies, including details of the tool(s) used, how many reviewers assessed each study and whether they worked independently, and if applicable, details of automation tools used in the process.	Page 19, 20
Effect measures	12	Specify for each outcome the effect measure(s) (e.g. risk ratio, mean difference) used in the synthesis or presentation of results.	Page 20
Synthesis methods	13a	Describe the processes used to decide which studies were eligible for each synthesis (e.g. tabulating the study intervention characteristics and comparing against the planned groups for each synthesis (item #5)).	Page 20
	13b	Describe any methods required to prepare the data for presentation or synthesis, such as handling of missing summary statistics, or data conversions.	NR
	13c	Describe any methods used to tabulate or visually display results of individual studies and syntheses.	Page 20
	13d	Describe any methods used to synthesize results and provide a rationale for the choice(s). If meta-analysis was performed, describe the model(s), method(s) to identify the presence and extent of statistical heterogeneity, and software package(s) used.	Page 20
	13e	Describe any methods used to explore possible causes of heterogeneity among study results (e.g. subgroup analysis, meta-regression).	Page 20
	13f	Describe any sensitivity analyses conducted to assess robustness of the synthesized results.	Page 20
Reporting bias assessment	14	Describe any methods used to assess risk of bias due to missing results in a synthesis (arising from reporting biases).	Page 19
Certainty assessment	15	Describe any methods used to assess certainty (or confidence) in the body of evidence for an outcome.	NR
**RESULTS**			
Study selection	16a	Describe the results of the search and selection process, from the number of records identified in the search to the number of studies included in the review, ideally using a flow diagram.	Page 4
	16b	Cite studies that might appear to meet the inclusion criteria, but which were excluded, and explain why they were excluded.	Page 4, 5
Study characteristics	17	Cite each included study and present its characteristics.	Page 4, 5
Risk of bias in studies	18	Present assessments of risk of bias for each included study.	Page 6
Results of individual studies	19	For all outcomes, present, for each study: (a) summary statistics for each group (where appropriate) and (b) an effect estimate and its precision (e.g. confidence/credible interval), ideally using structured tables or plots.	Page 6-9
Results of syntheses	20a	For each synthesis, briefly summarise the characteristics and risk of bias among contributing studies.	Page 6-9
	20b	Present results of all statistical syntheses conducted. If meta-analysis was done, present for each the summary estimate and its precision (e.g. confidence/credible interval) and measures of statistical heterogeneity. If comparing groups, describe the direction of the effect.	Page 6-9
	20c	Present results of all investigations of possible causes of heterogeneity among study results.	Page 6-9
	20d	Present results of all sensitivity analyses conducted to assess the robustness of the synthesized results.	Page 6-9
Reporting biases	21	Present assessments of risk of bias due to missing results (arising from reporting biases) for each synthesis assessed.	Page 6-9
Certainty of evidence	22	Present assessments of certainty (or confidence) in the body of evidence for each outcome assessed.	NR
**DISCUSSION**			
Discussion	23a	Provide a general interpretation of the results in the context of other evidence.	Page 9-15
	23b	Discuss any limitations of the evidence included in the review.	Page 14, 15
	23c	Discuss any limitations of the review processes used.	Page 14, 15
	23d	Discuss implications of the results for practice, policy, and future research.	Page 13, 14, 15
**OTHER INFORMATION**			
Registration and protocol	24a	Provide registration information for the review, including register name and registration number, or state that the review was not registered.	Page 16
	24b	Indicate where the review protocol can be accessed, or state that a protocol was not prepared.	Page 16
	24c	Describe and explain any amendments to information provided at registration or in the protocol.	NR
Support	25	Describe sources of financial or non-financial support for the review, and the role of the funders or sponsors in the review.	Page 20, 21
Competing interests	26	Declare any competing interests of review authors.	Page 20, 21
Availability of data, code and other materials	27	Report which of the following are publicly available and where they can be found: template data collection forms; data extracted from included studies; data used for all analyses; analytic code; any other materials used in the review.	Page 20, 21

From: Page MJ, McKenzie JE, Bossuyt PM, Boutron I, Hoffmann TC, Mulrow CD, et al. The PRISMA 2020 statement: an updated guideline for reporting systematic reviews. BMJ 2021;372:n71. doi: 10.1136/bmj.n71. This work is licensed under CC BY 4.0. To view a copy of this license, visit https://creativecommons.org/licenses/by/4.0/
